# An Integrated Cellular Computational Pipeline Decodes Luteolin to Design Possible Allosteric CDK1/CYCLIN B1 Inhibitors That Overcome Breast Cancer Stemness

**DOI:** 10.3390/ph19071048

**Published:** 2026-07-07

**Authors:** Rajesh Basnet, Buddha Bahadur Basnet, Muhammad Majid, Gogu Venkata Surendra Babu, Obed Boadi Amissah, Zhiyuan Li

**Affiliations:** 1 Guangzhou Institutes of Biomedicine and Health, Chinese Academy of Sciences, Guangzhou 510530, China; rajesh@gibh.ac.cn (R.B.); muhammad@gibh.ac.cn (M.M.); gogu.babu@gmail.com (G.V.S.B.); 2 University of Chinese Academy of Sciences, 19 Yuquan Road, Shijingshan District, Beijing 100049, China; 3 Department of Biomedical Science, Nepal Academy of Science and Technology, Lalitpur 44700, Nepal; budbsn.btechnep@gmail.com; 4 Departments of Immunology, Physiology and Biomedical Engineering, Mayo Clinic, Scottsdale, AZ 85259, USA; amissah.obed@mayo.edu; 5 Department of Anatomy & Neurobiology, Central South University, Changsha 410013, China

**Keywords:** luteolin (LT), HEK293T-GST-CDK1/CCNB1 cell, breast cancer stem cells, cell cycle regulation, anticancer and anti-stemness activity

## Abstract

**Background**: The dysregulation of the CDK1/Cyclin B1 complex drives tumor progression in breast cancer (BC). The natural flavonoid luteolin (LT) shows anti-cancer potential, but its mechanism targeting CDK1/CCNB1 remains unclear. **Methods**: *CDK1*, *CCNB1*, and *CCNB2* expression were profiled in normal and BC cell lines. An engineered HEK293T GST-CDK1/CCNB1 cell model was used to evaluate LT’s effects on proliferation, ROS levels, and target gene transcription. Computational approaches (molecular docking, dynamics simulations, pharmacophore modeling, MM/GBSA, ADMET, and network pharmacology) assessed LT and its analogues. **Results**: *CDK1/CCNB1* expression was lower in MCF7 BC cells than in normal cells, suggesting the loss of a growth barrier. In engineered HEK293T cells, LT suppressed *CCNB1* transcription with minimal effect on *CDK1* levels, correlating with anti-proliferative and ROS-modulating effects. Computational analyses confirmed stable LT binding to the CDK1/CCNB1 complex. Designed LT analogues showed improved binding and favorable ADMET profiles. Network pharmacology identified cell cycle regulation, particularly in BC stem cells, as the primary pathway targeted. **Conclusions**: LT and its analogues inhibit the CDK1/Cyclin B1 complex, revealing a dual mechanism that suppresses both tumor growth and BC stemness.

## 1. Introduction

Breast cancer (BC) remains a major cause of cancer related mortality, with dysregulation of cell cycle regulators driving tumor progression and stemness [1]. Despite advances in targeted therapies such as CDK4/6 inhibitors, treatment resistance and metastatic progression remain major clinical challenges, underscoring the urgent need for novel therapeutic strategies that target additional cell cycle nodes [2]. The CDK1/CCNB1 complex is a pivotal mediator of mitotic entry, and its aberrant activity has been implicated in ER + BC [3] and in aggressive subtypes including triple-negative BC (TNBC) [4]. A naturally occurring flavonoid present in a wide variety of fruits and vegetables, Luteolin (LT) has garnered significant interest due to its strong anticancer effects [5,6]. Natural compounds such as LT, a flavonoid abundant in fruits and vegetables, have attracted considerable attention for their ability to modulate oncogenic pathways with favorable safety profiles compared to conventional chemotherapeutics [6]. In addition to its antioxidant properties, LT interacts at the molecular level with cell cycle regulators, particularly cyclin-dependent kinases (CDKs), which are serine/threonine protein kinases essential for proper cell cycle progression [7]. Cyclins and CKIs regulate their actions, and disruption of this network is a key feature of CCDK1/CCNB which, importantly, remains underexplored as a direct molecular target of natural products compared to CDK4/6, which plays a central role in the G2/M phase transition [8]. Aberrant expression or mutation of CDK1 promotes unchecked proliferation in BCs [3]. As a result, CDK1 inhibition has emerged as a promising treatment option, with numerous small-molecule inhibitors advancing; however, optimisation remains difficult due to structural complexity and concerns about specificity. Moreover, no CDK1-specific inhibitor has yet achieved clinical approval, making the identification of selective CDK1-targeting scaffolds a high-priority area in drug discovery.

The anticancer activity of LT extends across diverse malignancies, notably models of breast (MDA-MB-231), colon (HCT116), gastric (MKN45, BGC823), prostate (PC-3, DU145), and lung cancers [9]. Mechanistically, LT downregulates key oncogenic drivers, including PD-L1 and cyclin D1, while upregulating the cell-cycle inhibitor p21/WAF1. Additionally, it modulates several signaling pathways, including Notch1, PI3K/AKT/mTOR, ERK, and STAT3, and inhibits the phosphorylation of IGF-1R and PDGF receptor, thereby reinforcing its broad chemoprotective profile [9]. However, these prior studies have largely focused on indirect or downstream effects, leaving a critical gap: whether LT directly binds to and inhibits the CDK1/CCNB1 holoenzyme remains unknown. Despite LT’s has broad biological effects, its direct molecular interaction with the CDK1/CCNB1 complex and its role in cell cycle regulation remain insufficiently defined. To address this, we engineered an HEK293T-GST-CDK1/CCNB1 cell line to directly examine LT–CDK1/CCNB1 interactions. Using this model, we assessed transcriptional changes in *CDK1*, *CCNB1*, and *CCNB2*, along with phenotypic outcomes, including modulation of ROS levels. These approaches revealed the functional roles of CDK1, CCNB1, and CCNB2 in LT-mediated anti-proliferative effects, underscoring LT’s potent dual anticancer and anti-stemness activities [10]. Furthermore, we characterized the binding affinities and molecular dynamics of the LT. On the basis of these, we evaluated previously reported LT analogues as potential CDK1/CCNB1 inhibitors *via* molecular docking, molecular dynamics simulations, MM/GBSA calculations, ADMET profiling, and network pharmacology. This integrated computational and experimental approach allowed us to prioritize LT analogues with improved predicted bioavailability and CDK1/CCNB1 binding selectivity over earlier natural product derivatives. Our work predicts the rational design of bioavailable LT analogues that inhibit BC by targeting the CDK1/CCNB1 complex, uncovering a non-canonical redox–transcriptional axis controlling this target and providing a preclinical framework for developing LT-based CDK1 inhibitors with translational potential.

## 2. Results

### 2.1. RNA-Seq Reveals Elevated CDK1/CCNB Complex Expression in Non-Tumorigenic Cell Lines, Defining a Proliferative Barrier Absent in MCF7 Breast Cancer Cells

**Figure 1:**
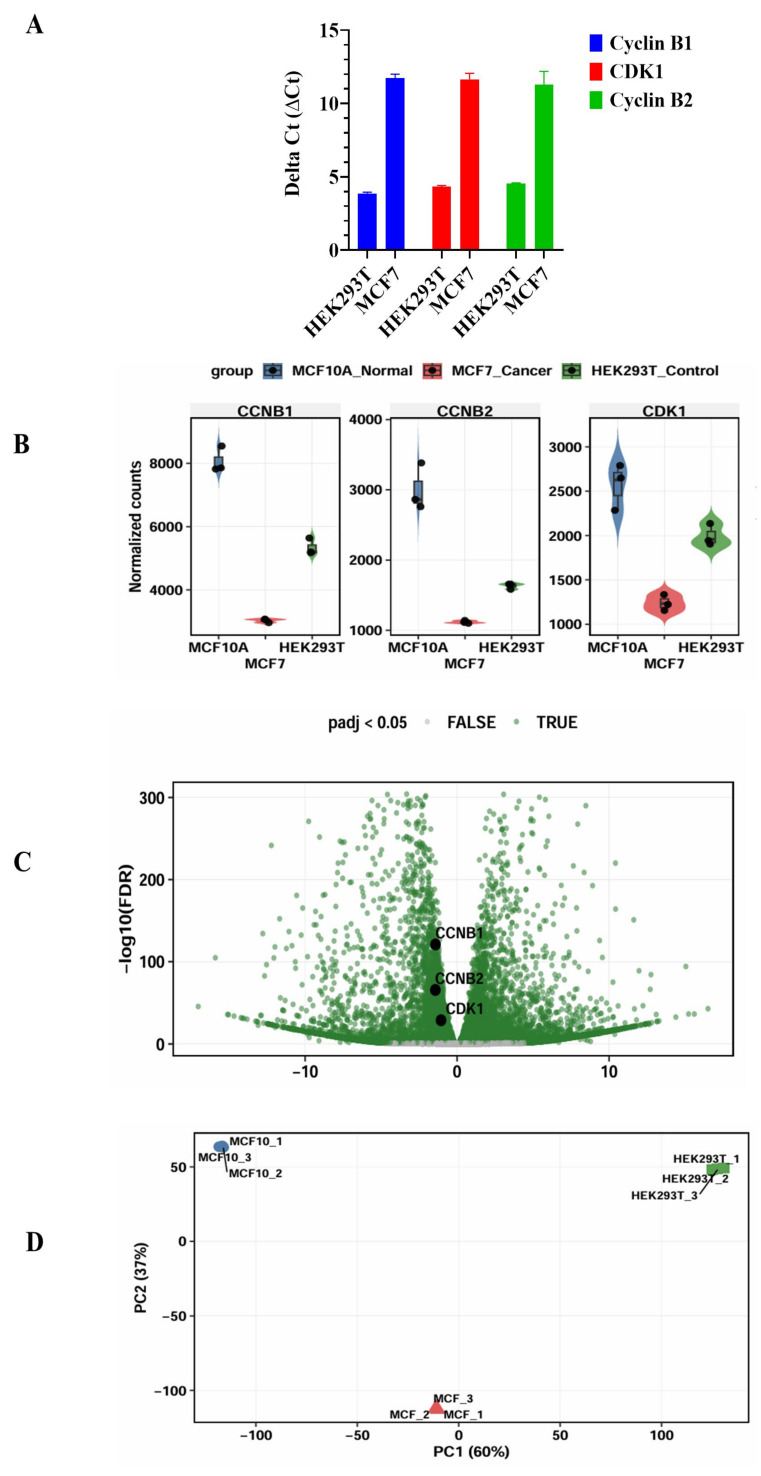

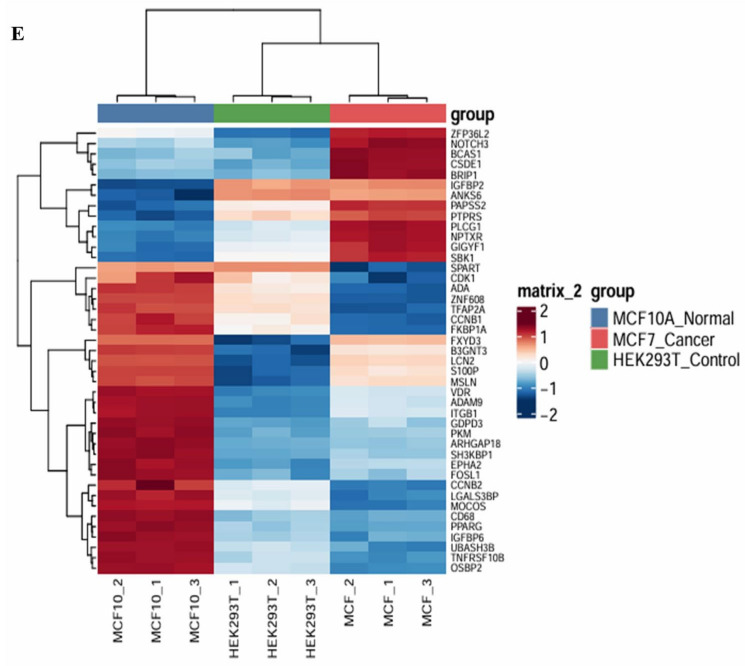
Differential gene expression profiles of *CDK1*, *CCNB1*, and *CCNB2* across MCF7 (cancer), MCF10A (normal), and HEK293T (control) cell lines. (**A**) Delta Ct (ΔCt) values of *CDK1*, *CCNB1*, and *CCNB2* genes in MCF7 compared with HEK293T cells as a control. The results represent three independent experiments, shown as mean ± S.D. (**B**) Expression analysis of *CDK1*, *CCNB1*, and *CCNB2* in MCF7, MCF10A, and HEK293T cells. (**C**) Volcano plot depicting significantly dysregulated genes in MCF7 relative to MCF10A. (**D**) Principal component analysis (PCA) plot showing distinct clustering of MCF7, MCF10A, and HEK293T samples. (**E**) Heatmap displaying the top differentially expressed genes with log_2_ fold change values for MCF7 relative to MCF10A.

The ΔCt values for *CDK1*, *CCNB1*, and *CCNB2* were lower in HEK293T cells compared to the MCF7 BC cell line, indicating higher expression levels in HEK293T cells (Figure 1A), as ΔCt values are inversely proportional to gene expression [11], highlighting cell line-specific transcriptional differences between breast epithelial and embryonic kidney cells. Consistently, RNA Seq analysis confirmed that these genes are more highly expressed in MCF10A (normal) and HEK293T (control) cells than in MCF7 (Figure 1B). Throughout this study, absolute expression comparisons are made relative to MCF7 as the reference unless otherwise specified. Differential gene expression analysis between MCF7 and MCF10A revealed multiple significantly dysregulated genes, including *CCNB1*, *CCNB2*, and *CDK1*. However, as shown in Figure 1B, absolute expression levels of these genes are higher in MCF10A and HEK293T, and lowest in MCF7. The positive fold change in Figure 1C therefore reflects a pairwise comparison between MCF7 and MCF10A only and does not indicate that MCF7 has the highest absolute expression across all three cell lines, implicating their role in cell cycle regulation and BC progression (Figure 1C). PCA analysis showed clear separation among MCF10A, MCF7, and HEK293T samples, with PC1 and PC2 accounting for 60% and 37% of the variance, respectively, indicating distinct transcriptomic profiles (Figure 1D). In Figure 1E, hierarchical clustering of log2 fold change values revealed distinct grouping of samples and genes, with red and blue gradients indicating upregulation and downregulation, respectively. Notably, genes such as ZFP36L2, NOTCH3, and BRIP1 displayed marked differential expression across cell types. In addition, clustering analysis highlighted distinct expression profiles of *CDK1*, *CCNB1*, and *CCNB2*. RNA-Seq data revealed higher expression levels of these genes in HEK293T and MCF10A cells compared to MCF7 cells, consistent with *in vitro* findings. Taken together, the transcriptomic profiles support the conclusion that *CDK1*, *CCNB1*, and *CCNB2* expression is higher in MCF10A and HEK293T, and lowest in MCF7. This aligns with the *in vitro* data and underscores the critical role of these genes in cell cycle progression and BC.

### 2.2. Establishment and Validation of an HEK293T Cell Line Expressing GST-Tagged CDK1/CCNB

**Figure 2:**
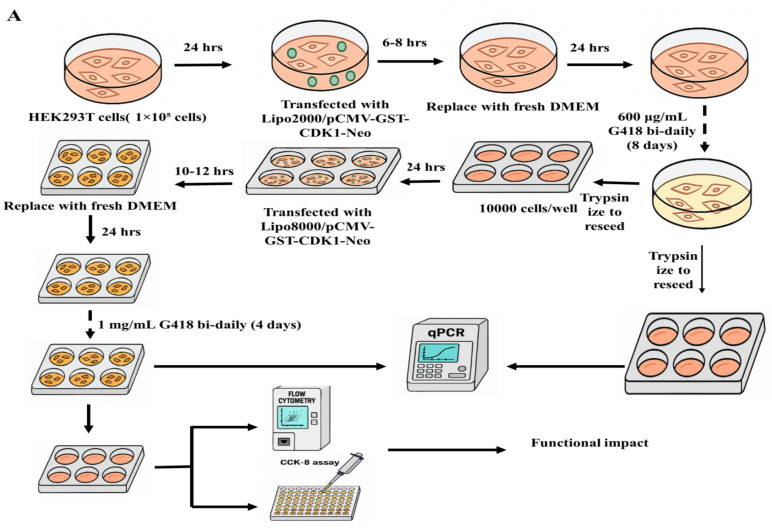

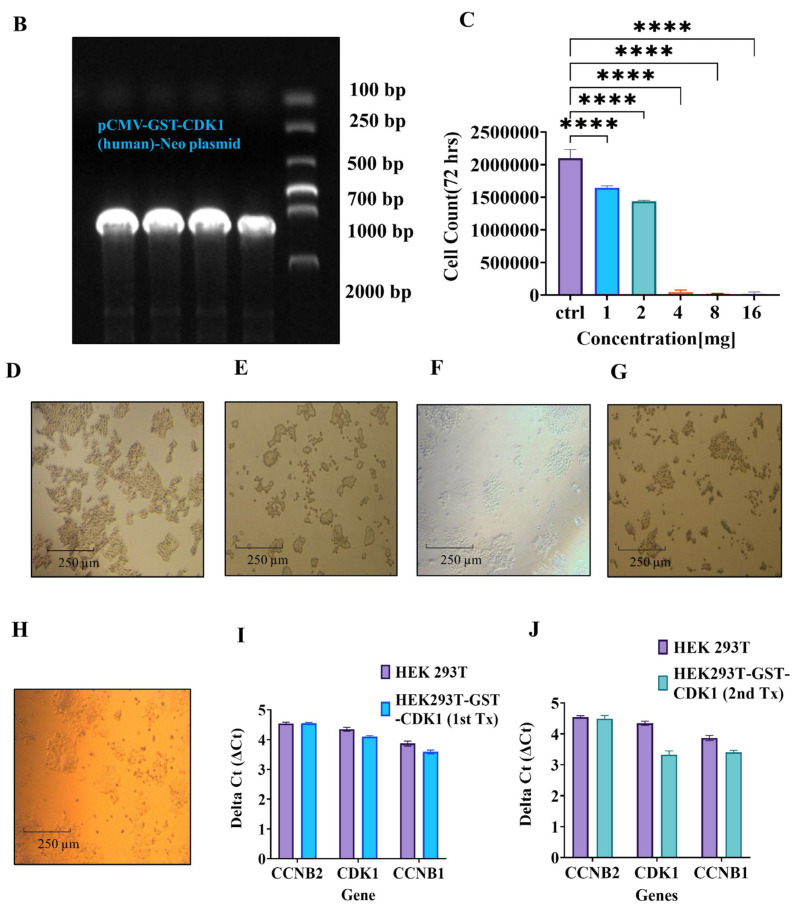
Verification of the pCMV-GST-CDK1 (human)-Neo plasmid and establishment of the HEK293T-GST-CDK1/CCNB1 cell line. (**A**) Overview of the procedure applied to HEK293T cells, which were transfected with the pCMV-GST-CDK1 (human)-Neo plasmid to induce gene expression. (**B**) PCR amplification of the pCMV-GST-CDK1 (human)-Neo plasmid using gene-specific primers and 2 × Taq PCR StarMix (Dye). Lane 1: 2 kb DNA ladder; Lanes 2–5: plasmid samples showing expected CDK1 band sizes, confirming plasmid integrity. Microscopic images of HEK293T cells. (**C**) Optimisation of G418 effects on HEK293T cell viability during transfection. (**D**) Untransfected control cells. (**E**) HEK293T cells transfected with pCMV-GST-CDK1 and selected with G418 (0 days). (**F**) HEK293T cells transfected with pCMV-GST-CDK1 and G418-selected (8 days). (**G**) G418-selected cells re-transfected with pCMV-GST-CDK1 (0 days). (**H**) G418-selected HEK293T cells re-transfected with pCMV-GST-CDK1 and G418-selected (4 days). (**I**) Quantitative PCR analysis showing ΔCt values in HEK293T-GST-CDK1/CCNB1 cells compared to non-transfected controls, validating stable integration and expression after the first transfections. (**J**) Quantitative PCR analysis showing ΔCt values in HEK293T-GST-CDK1/CCNB cells compared to non-transfected controls, validating stable integration and expression after the second transfections. The results represent three independent experiments and are presented as mean ± SD (**** *p* < 0.0001 vs. control).

HEK293T cells were selected for overexpression studies because they exhibit exceptionally high transfection efficiency, driven by the presence of the SV40 large T antigen [12], enabling robust plasmid replication and strong gene expression of *CDK1*, *CCNB1*, and *CCNB2*. In order to generate a cell line expressing GST tagged CDK1, the pCMV-GST-CDK1 (human)-Neo plasmid was introduced into DH5α competent cells *via* heat shock transformation and selected on ampicillin-containing agar plates. Figure 2A provides an overview of the procedure applied to HEK293T cells, which were transfected with the pCMV-GST-CDK1 (human) Neo plasmid to induce gene expression. Following plasmid amplification and purification, PCR analysis confirmed plasmid integrity by showing the expected CDK1 band sizes (Figure 2B). HEK293T cells were initially transfected with Lipofectamine 2000 and incubated for 48 h. Optimisation of G418 effects on cell viability during transfection is shown in Figure 2C, and untransfected control cells are presented in Figure 2D. Transfected HEK293T cells were subjected to 600 μg/mL G418 selection for 8 days, followed by a second transfection using Lipofectamine 8000 to enhance *CDK1*, *CCNB1* and *CCNB2* expression further. Subsequent selection with 1 mg/mL G418 for 4 days enriched for cells expressing the GST-tagged CDK/CCNB fusion protein. Representative images of both the first and second transfections are shown in Figure 2E–H. Quantitative PCR analysis indicated that the ΔCt values for *CDK1* and *CCNB1* were significantly lower in twice-transfected HEK293T cells than in parental cells, indicating elevated transcript levels (Figure 2I,J). In contrast, no change in *CCNB2* expression was observed. Luteolin (Catalogue #CFN98784, ChemFaces was purchased for an *in vitro* study to assess its functional impact on HEK293T cells and twice-transfected HEK293T cells.

### 2.3. Luteolin Triggers Oxidative Stress to Suppress CDK1/CCNB1 Activity and Expression, Driving Morphological and Proliferative Changes in Expressed HEK293T-GST-CDK1/CCNB1 Cells

**Figure 3:**
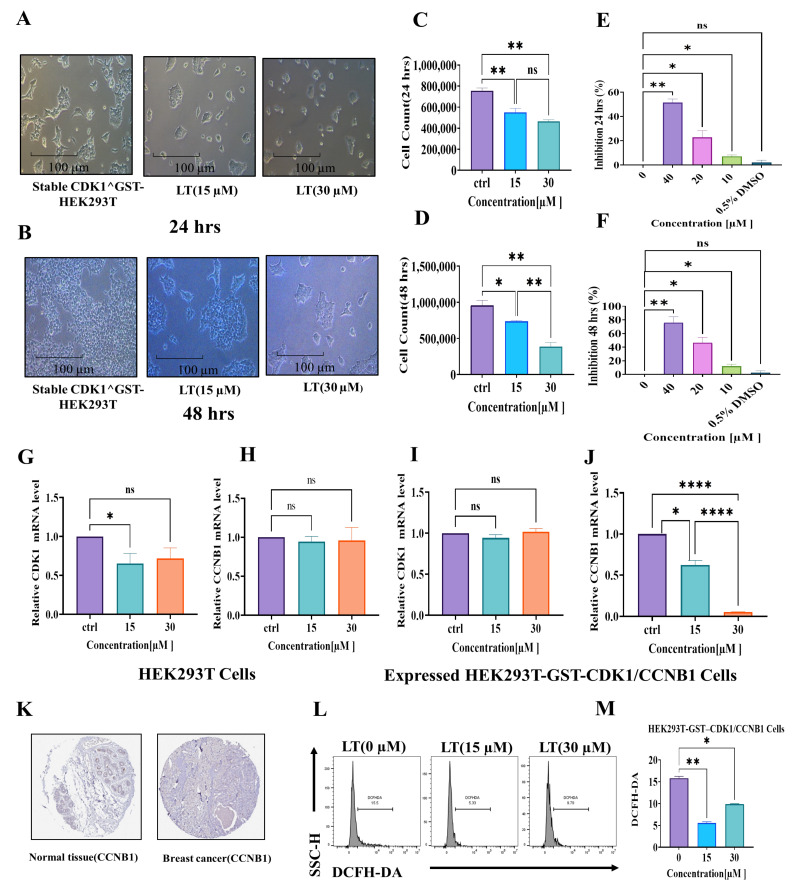
Effects of LT treatment over time in HEK293T-GST-CDK1/CCNB1 cells, and relative expression of *CDK1* and *CCNB1* in HEK293T and HEK293T-GST-CDK1/CCNB1 cells. (**A**) Representative microscopic images of HEK293T-GST-CDK1/CCNB1 cells after 24 h of LT treatment. (**B**) Representative microscopic images of HEK293T-GST-CDK1/CCNB1 cells after 48 h of LT treatment. (**C**) Quantification of cell counts per mL at 24 h post-treatment. (**D**) Quantification of cell counts per mL at 48 h post-treatment. (**E**) Cell viability inhibition assays using CCK-8 at 24 h. (**F**) Cell viability inhibition assays using CCK-8 at 48 h. (**G**) Fold changes in *CCNB1* expression between DMSO-treated controls and LT-treated HEK293T cells. (**H**) Fold changes in *CDK1* expression between DMSO-treated controls and LT-treated HEK293T cells. (**I**) Fold changes in *CCNB1* expression between DMSO-treated controls and LT-treated HEK293T-GST-CDK1/CCNB1 cells. (**J**) Fold changes in *CDK1* expression between DMSO-treated controls and LT-treated HEK293T-GST-CDK1/CCNB1 cells. (**K**) Immunohistochemical data from the HPA database showing higher CCNB1 expression in breast cancer tissues compared with normal breast tissues. (**L**) Representative histograms of ROS levels in untreated control cells and HEK293T-GST-CDK1/CCNB1 cells treated with 15 μM and 30 μM LT for 48 h, measured by DCFH-DA staining. (**M**) Quantification of ROS detection. The results represent three independent experiments and are presented as mean ± SD (* *p* < 0.05, ** *p* < 0.01, and **** *p* < 0.0001 vs. control).

The HEK293T-GST-CDK1/CCNB1 cell line exhibited healthy morphology and a regular proliferation rate. It is important to clarify that this model involves artificial overexpression of *CDK1* and *CCNB1* in a non-cancerous background, so the expression levels observed here do not represent their physiological status in normal breast tissue. Treatment with 15 μM LT-induced visible morphological changes, reduced cell density, and signs of cellular stress. These effects were more pronounced at 30 μM LT, including marked cell shrinkage and significantly decreased proliferation at both 24 h and 48 h (Figure 3A,B). Consistently, the number of cells decreased at both time points (Figure 3C,D). Cells were exposed to increasing concentrations of LT (0–40 μM) for 24 and 48 h. LT treatment significantly inhibited the growth of HEK293T-GST-CDK1/CCNB1 cells (Figure 3E,F). Still, in the HEK293T parental cells, it had no significant effect on cell viability, as we reported previously [10], suggesting that the overexpressed *CDK1* and *CCNB1* were functionally responsive to LT. To further explore this responsiveness, HEK293T-GST-CDK1/CCNB1 cells were treated with 15 μM and 30 μM LT, and gene expression changes were assessed. HEK293T parental cells showed no or minimal changes in *CDK1* and *CCNB1* transcript levels following LT treatment (Figure 3G,H). In contrast, in HEK293T-GST-CDK1/CCNB1 cells, LT treatment led to apparent downregulation of *CCNB1* and only a minimal impact on *CDK1* transcript levels (Figure 3I,J). To determine *CDK1* and *CCNB1* expression in BC, these genes were first analysed in clinical specimens from the HPA database. In contrast to the overexpression system, analysis of clinical specimens revealed that *CDK1* and *CCNB1* are highly expressed in BC tissues, whereas their expression is low in normal breast tissue (Figure 3K). This finding confirms that both genes are indeed upregulated in BC, supporting their relevance as potential therapeutic targets. To evaluate the effect of LT on cellular ROS levels, we quantified ROS in LT-treated cells using DCFH-DA staining. Our results showed that LT treatment significantly reduced ROS levels (greater reduction at 15 μM than at 30 μM; Figure 3L,M). Given that *CDK1* and *CCNB1* are naturally upregulated in BC (Figure 3K), the observed ROS reduction in our overexpression model suggests that LT may exert its anticancer effects, at least in part, by modulating cellular redox balance. This reduction in oxidative stress, potentially linked to CDK1/CCNB1 inhibition, may contribute to LT’s anticancer activity by modulating redox balance in HEK293T-GST-CDK1/CCNB1 cells.

### 2.4. Molecular Docking and Dynamics Simulations Reveal Luteolin’s a Possible Allosteric Inhibition of the CDK1/CCNB1 Cell-Cycle Complex

**Figure 4:**
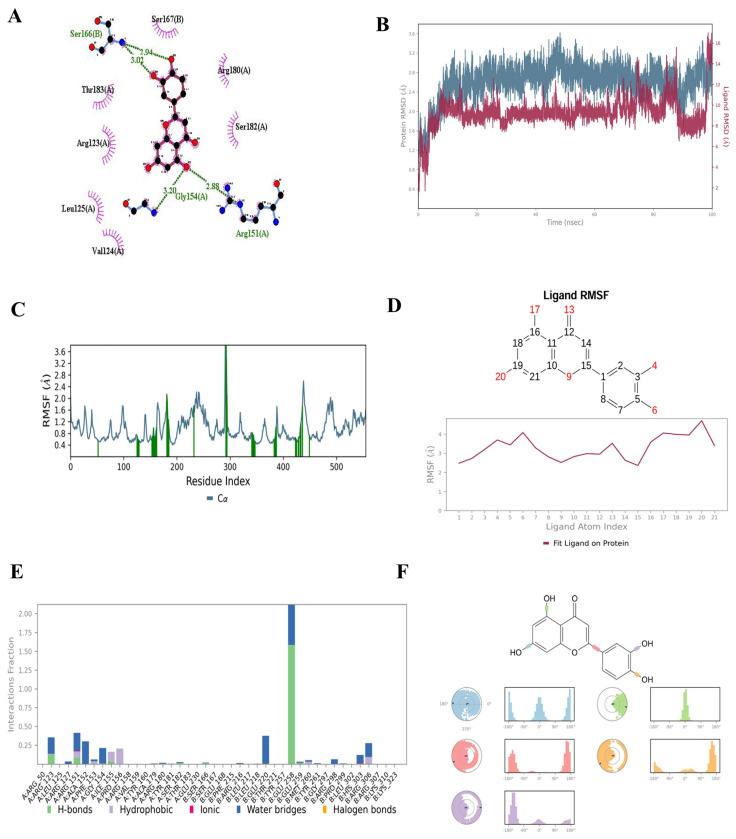
Molecular docking and dynamics simulation of LT with the CDK1/CCNB1 cell cycle complex, illustrating LT targeting of the CDK1/CCNB1 regulatory network in BC. (**A**) Binding interactions of LT with three key residues within the CDK1/CCNB1 complex. (**B**) RMSD analysis of the CDK1/CCNB1 complex in the presence of LT. (**C**) RMSF plots showing protein backbone fluctuations (blue) and LT ligand contacts (green). (**D**) RMSF plots highlighting ligand flexibility during the simulation. (**E**) Contact map of CDK1/CCNB1–LT interactions over the simulation trajectory. (**F**) Torsion profile of LT illustrating conformational changes during binding.

Molecular docking and dynamics simulations were performed to investigate LT interactions with the CDK1/CCNB1 cell cycle complex under the applied computational protocol. As shown in Figure 4A, LT formed binding interactions with key residues within the complex. RMSD analysis (Figure 4B) revealed that the protein backbone remained stable throughout the 100 ns simulation, fluctuating modestly between 2.0 and 2.5 Å. At the same time, the ligand exhibited a higher RMSD of approximately 14 Å, suggesting that the ligand deviated from its initial docked pose during the simulation. RMSF plots (Figure 4C) showed low fluctuations (<2 Å) for most protein residues, consistent with structural rigidity, with localized peaks (~3.6 Å) corresponding to loop regions or termini. Ligand RMSF analysis highlighted atom-specific flexibility, with atoms 9, 13, 17, and 20 showing the highest fluctuations (~5 Å), which may indicate that the ligand drifted out of the binding pocket during the simulation (Figure 4D). The contact map (Figure 4E) showed initial LT–CDK1/CCNB1 interactions that diminished over time, while the torsion profile (Figure 4F) illustrated conformational changes in LT during the simulation. Collectively, these observations are consistent with the CDK1/CCNB1 complex maintaining structural stability throughout the 100 ns simulation, whereas LT binding appeared to be unstable under the conditions tested. We note that a ligand RMSD of approximately 14 Å is relatively high and suggests that the ligand did not maintain a stable binding pose during the simulation. This may indicate that the ligand drifted out of the predicted binding pocket, which would not be consistent with a model of stable, sustained binding. Therefore, our computational findings should be interpreted as suggestive of weak or transient interactions, and further experimental studies would be required to confirm functional binding. Overall, molecular docking and dynamics simulations are consistent with a potential possible allosteric inhibitor mode of the CDK1/CCNB1 complex; however, the high ligand mobility observed raises questions about the stability of this binding mode under the simulation conditions.

### 2.5. Network Analysis Identifies a Critical Regulatory Hub at the CDK1/CCNB1 Complex Interface

**Figure 5:**
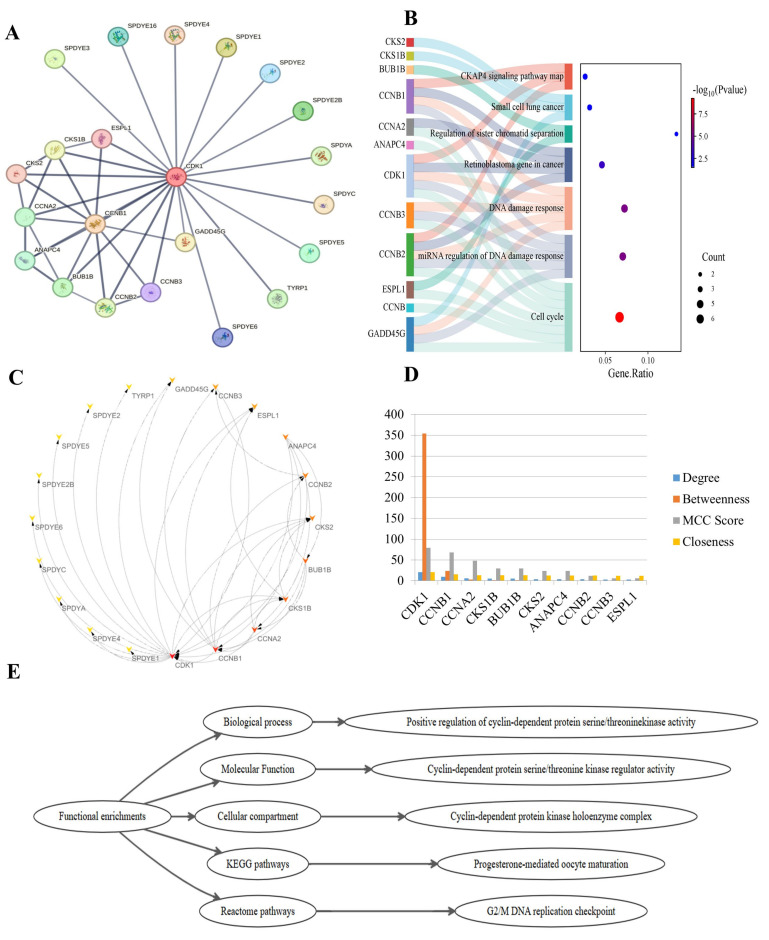
Network analysis CDK1/CCNB1 complex. (**A**) STRING derived PPI network showing CDK1 and CCNB1 as central nodes, connected to key mitotic regulators. Edge thickness reflects interaction confidence (score ≥ 0.7). (**B**) Wikipathway enrichment analysis highlighting significant involvement in the cell cycle. (**C**) Circular layout of the top 20 genes ranked by degree centrality within the CDK1/CCNB1 interaction network. (**D**) Top-ranked hub proteins in the CDK1/CCNB1 complex network (cytoHubba analysis). Degree represents the number of direct connections; Betweenness indicates control over information flow; MCC (Maximal Clique Centrality) measures robustness within cliques; Closeness reflects the efficiency of a node in reaching all other nodes in the network (inverse of average shortest path length). (**E**) Top Functional enrichment of CDK1/CCNB1 complex.

The STRING derived protein–protein interaction (PPI) network revealed CDK1 and CCNB1 as central hub nodes, tightly interconnected with key mitotic regulators. Edge thickness in the network reflects interaction confidence, with scores ≥ 0.7 indicating strong associations (Figure 5A). Subsequent Wikipathway enrichment analysis highlighted a significant role for these nodes in cell cycle regulation, underscoring their functional relevance and supporting the hypothesis that LT analogues may exert anti-cancer effects by modulating CDK1/CCNB1-mediated pathways (Figure 5B). In Figure 5C, the circular layout illustrates the top 20 genes ranked by degree centrality within the CDK1/CCNB1 interaction network. CDK1 and CCNB1 emerge as the most dominant nodes, exhibiting the highest degree, betweenness, MCC, and closeness centrality values, as summarised in Figure 5D. Functional enrichment analysis (Biological Process, Molecular Function, Cellular Compartment, KEGG, Reactome) confirms the CDK1/CCNB1 complex as a central regulator of the cell cycle in Figure 5E. This underscores their pivotal roles in the network’s regulatory architecture.

### 2.6. Rational Design and Multi-Parameter Optimisation Yield Bioavailable Luteolin Analogues Predicted to Inhibit Breast Cancer

**Names and their SMILES:-**

(i) 2-(3,4-dihydroxyphenyl)-7-hydroxy-4-oxo-4H-chromen-5-yl acetate

SMILE: O=C1C2=C(OC(C)=O)C=C(O)C=C2OC(C3=CC=C(O)C(O)=C3)=C1

(ii) 2-(3,4-dihydroxyphenyl)-7-hydroxy-4-oxo-4H-chromen-5-yl methyl succinate

SMILE: O=C1C2=C(OC(CCC(OC)=O)=O)C=C(O)C=C2OC(C3=CC=C(O)C(O)=C3)=C1

(iii) 2-(3,4-dihydroxyphenyl)-7-hydroxy-4-oxo-4H-chromen-5-yl benzoate

SMILE: O=C1C2=C(OC(C3=CC=CC=C3)=O)C=C(O)C=C2OC(C4=CC=C(O)C(O)=C4)=C1

(iv) 2-(3,4-dihydroxyphenyl)-7-hydroxy-4-oxo-4H-chromen-5-yl 2-phenylacetate

SMILE: O=C1C2=C(OC(CC3=CC=CC=C3)=O)C=C(O)C=C2OC(C4=CC=C(O)C(O)=C4)=C1

(v) 2-(3,4-dihydroxyphenyl)-5,7-dihydroxy-4H-chromene-4-thione

SMILE: S=C1C2=C(O)C=C(O)C=C2OC(C3=CC=C(O)C(O)=C3)=C1

(vi) 4-(7-acetoxy-5-hydroxy-4-oxo-4H-chromen-2-yl)-1,2-phenylene diacetate

SMILE: O=C1C2=C(O)C=C(OC(C)=O)C=C2OC(C3=CC=C(OC(C)=O)C(OC(C)=O)=C3)=C1

(vii) 4-(5,7-diacetoxy-4-oxo-4H-chromen-2-yl)-1,2-phenylene diacetate

SMILE:O=C1C2=C(OC(C)=O)C=C(OC(C)=O)C=C2OC(C3=CC=C(OC(C)=O)C(OC(C)=O)=C3)=C1

(viii) 4-(5-hydroxy-4-oxo-7-(propionyloxy)-4H-chromen-2-yl)-1,2-phenylene dipropionate

SMILE:O=C1C2=C(O)C=C(OC(CC)=O)C=C2OC(C3=CC=C(OC(CC)=O)C(OC(CC)=O)=C3)=C1

**Figure 6:**
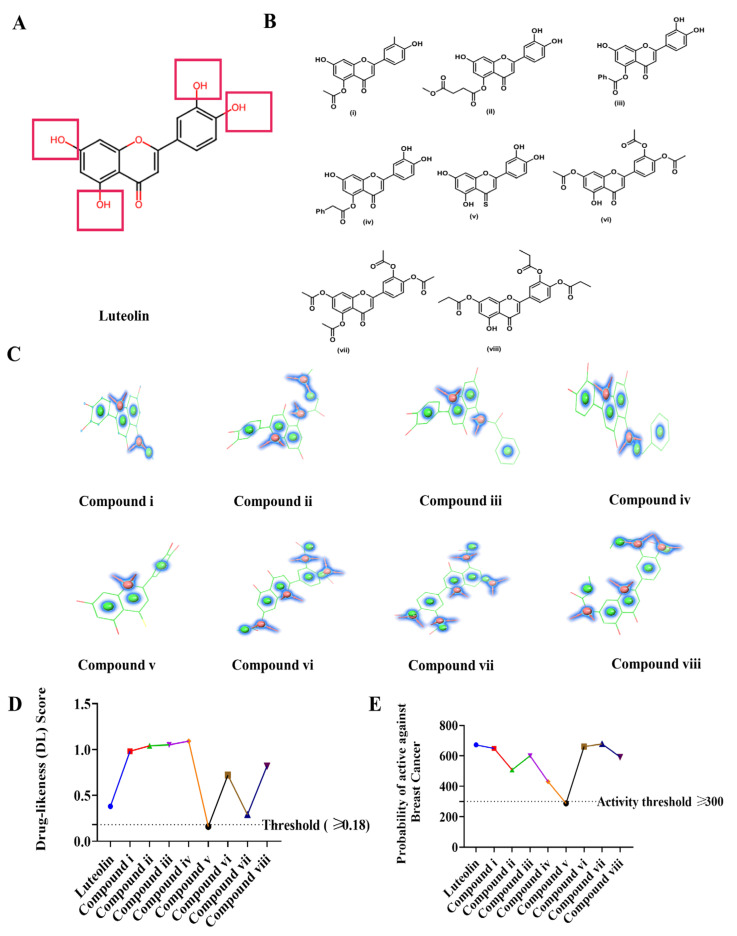
Rational design and multi-parameter optimization of LT analogues. (**A**) LT flavone core structure and key hydroxyl group positions (**B**). Chemical structures of different LT analogues using 5-O-acetyl luteolin as the core structure. (**C**) Pharmacophore analysis of different LT analogues shows the strongest expression of key features (H-bond acceptor, A; hydrophobic region, H). (**D**) A line plot showing Drug-likeness scores from the MolSoft tool for LT analogues. (**E**) A line plot showing favourable predicted activity against BC scores from the PASS Online tool for LT analogues.

The biological activities of LT arise from its flavone core substituted with hydroxyl groups at the C3, C4, C5, and C7 positions (Figure 6A). A series of eight structural analogues was designed based on the LT scaffold. Using 5-O-acetyl luteolin as the core structure, systematic modifications including acetylation, esterification, and heteroatom substitution were performed. The selection of these specific analogues was guided by prior evidence of pharmacological activity, with the introduced variations, such as hydroxyl group substitutions and ring modifications, anticipated to modulate affinity and binding orientation within the target’s active site [13]. All designed LT analogues were validated for chemical correctness using Open Babel 3.1.1. Their key physicochemical properties were calculated with RDKit 2021.09.4 (Figure 6B). Subsequent pharmacophore analysis of the designed analogues identified that the key features, hydrogen bond acceptors (A) and hydrophobic regions (H), were most strongly expressed in compounds vii and viii. This result indicates that these two analogues exhibit the closest alignment with the essential interaction points of the pharmacophore model (Figure 6C). Following pharmacophore analysis, the drug-likeness (DL) of all LT analogues was assessed. Compounds were filtered using a DL score threshold of ≥0.18, as implemented in the SwissADME web tool [14]. Seven of the eight analogues met this criterion; however, analogue v fell below this threshold, indicating potential liabilities for its further development as an oral drug candidate (Figure 6D). All designed analogues exhibited probability activity of activity (Pa ≥ 300) against BC cells [15], with the notable exception of compound v (Figure 6E). Finally, through rational design and multiparameter optimization, we have developed a series of bioavailable LT analogues predicted to show enhanced efficacy against BC.

### 2.7. Luteolin Analogues Exhibit CDK1/CCNB1 Binding Mechanisms Distinct from the Parent Scaffold

**Figure 7:**
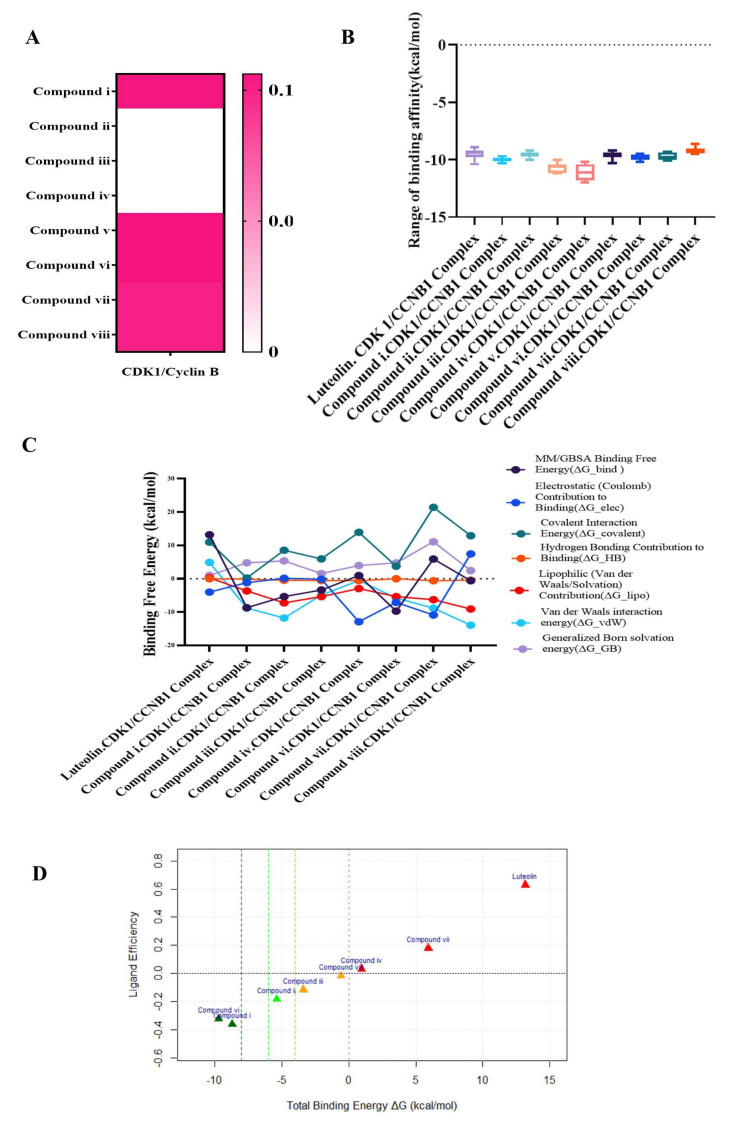

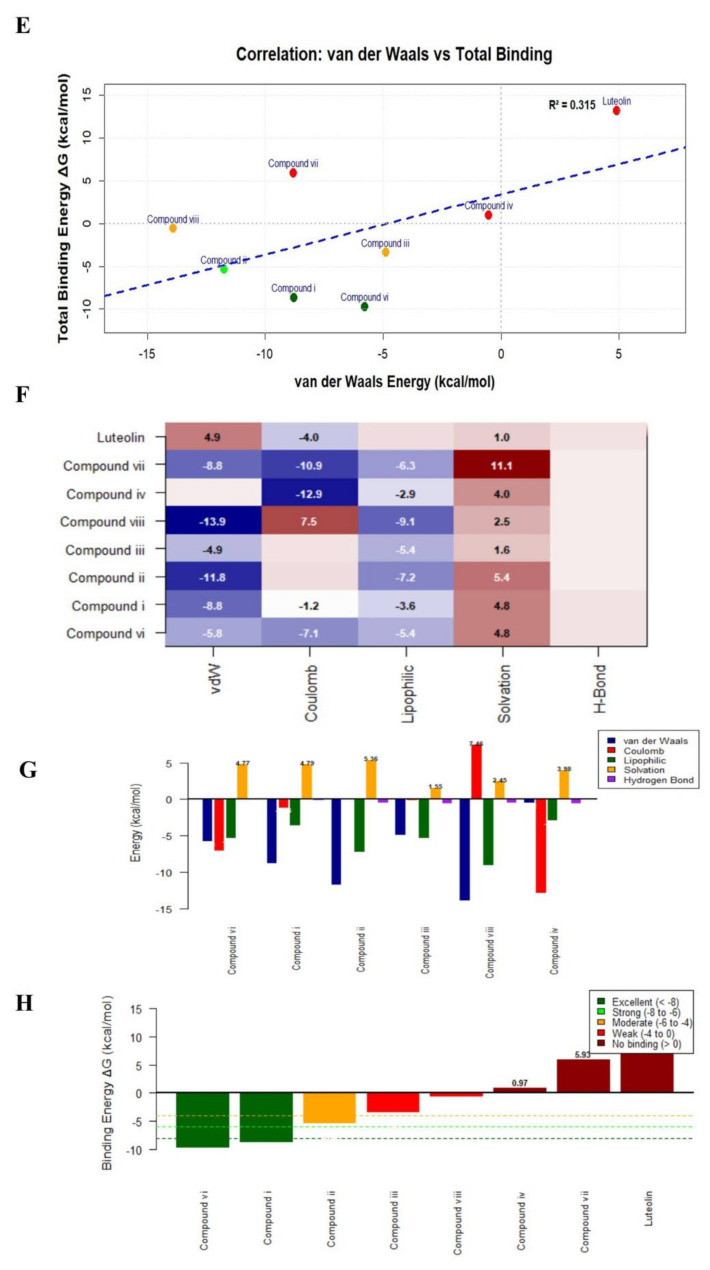
Computational strategy for evaluating LT analogues as CDK1/CCNB1 inhibitors. (**A**) Heat map showing the probability values of LT analogues predicted to target the CDK1/CCNB complex using SwissTargetPrediction. (**B**) Range of binding affinities (kcal/mol) illustrating the distribution of docking scores for LT and its analogues against the CDK1/CCNB1 complex. (**C**) Decomposition of MM/GBSA binding free energies (ΔG_bind) for LT analogues to the CDK1/CCNB1 complex. (**D**) Ligand efficiency (ΔG/heavy atom) versus binding energy landscape (**E**) Correlation between van der Waals energy and total binding (R^2^ = 0.315). (**F**) Heatmap of energy components across all analogues (blue: favourable, red: unfavourable). (**G**) Energy decomposition for the top five binders showing van der Waals (blue), Coulombic (red), lipophilic (green), solvation (orange), and hydrogen bonding (purple) contributions. (**H**) Total binding energies (ΔG, kcal/mol) from MMGBSA calculations. Compounds ranked by binding affinity with colour indicating strength: dark green (excellent, ΔG < −8), green (strong, −8 to −6), orange (moderate, −6 to −4), red (weak, −4 to 0), dark red (no binding, >0) and LT reference shown as a purple dashed line.

To investigate a potential mechanistic basis, we employed SwissTargetPrediction to identify probable protein targets for CDK1/CCNB complex. The resulting ligand based similarity scores, visualised in a heat map, indicate the relative probability of each analogue interacting with the CDK1/CCNB complex. Notably, analogues ii, iii, and iv showed lower predicted affinity for this complex, while all others, including the highly active compounds, displayed a high predicted affinity (Figure 7A). Notably, molecular docking predicted that several LT analogues bind more strongly than LT, with affinities ranging from −9 to −12 kcal/mol, suggesting these compounds achieve superior stabilization in the CDK1/CCNB1 binding pocket (Figure 7B). MM/GBSA calculations across eight LT analogues consistently predicted favourable binding to the CDK1/CCNB1 complex, with ΔG_bind values ranging from moderately negative to low. Energy decomposition analysis revealed that binding was primarily driven by electrostatic stabilisation (ΔG_elec), which was partially offset by desolvation penalties (ΔG_GB) (Figure 7C). Among the series, Compound v was not further studied for binding mechanisms due to its poor drug-likeness (DL score < 0.18) and low predicted activity (Pa < 300) against BC cells. Compound vi demonstrated strong binding to the CDK1/CCNB1 complex, and most tested compounds showed better ligand efficiency than LT (Figure 7D). Van der Waals interactions showed a positive but weak correlation with total binding affinity (R^2^ = 0.315), indicating that other energy contributions also play important roles (Figure 7E). At the same time, energy component heatmaps revealed consistent patterns of favorable van der Waals and lipophilic interactions with variable coulombic and solvation contributions across the LT analogue series (Figure 7F). Energy decomposition confirmed that both van der Waals and Coulomb interactions drive binding, with their relative contributions varying across top binders (Figure 7G). MMGBSA analysis revealed that compounds vi, i, and ii exhibited enhanced binding to the CDK1/CCNB1 complex compared to the parent LT, with compound vi showing the strongest affinity (ΔG = −9.72 kcal/mol) (Figure 7H). Notably, structural modifications to the LT scaffold generated analogues that engage the CDK1/CCNB1 complex *via* distinct binding mechanisms, with significantly modulated affinity, highlighting several promising candidates for experimental validation in ER^+^ BC models.

### 2.8. Molecular Docking of Luteolin Analogues Reveals a Conserved Binding Mode Targeting Critical CDK1/CCNB1 Interface Residues

**Figure 8:**
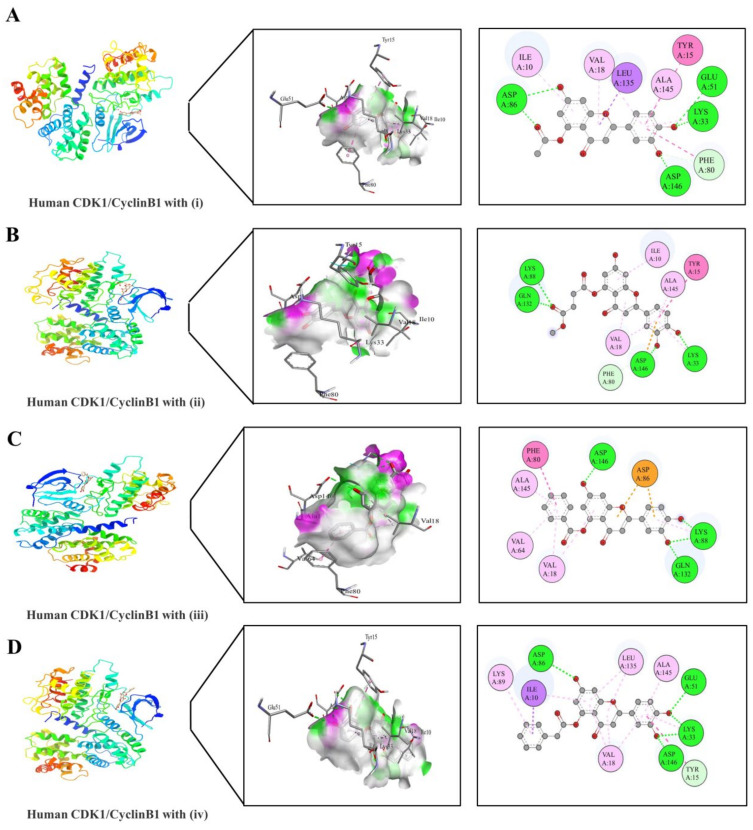

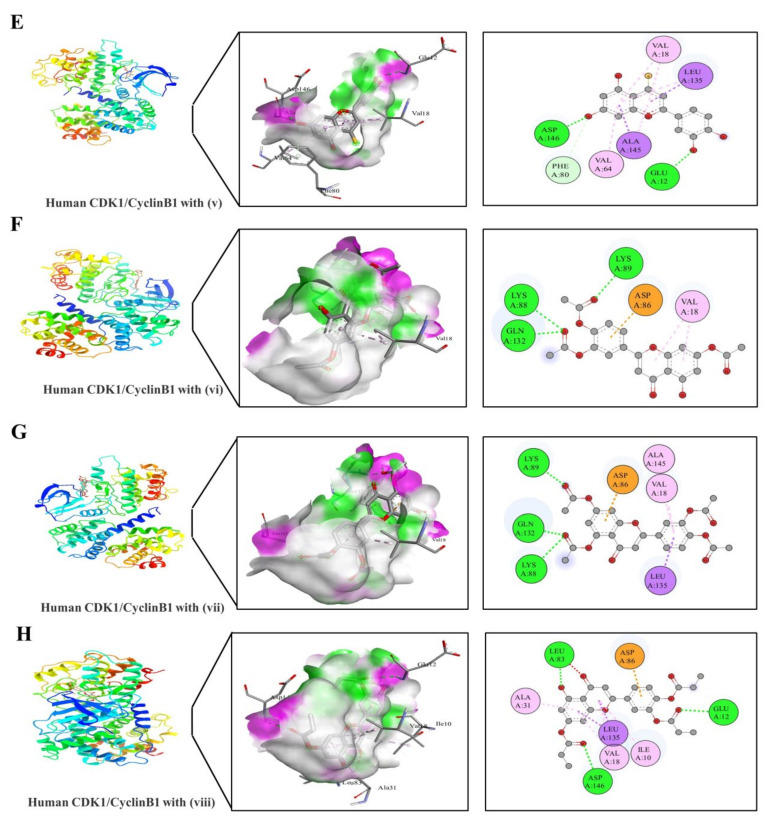
Visualising LT analogous molecular docking interaction with the CDK1/CCNB1 complex using Discovery Studio 2021 (2D Structure) and UCSF ChimeraX (Surface). (**A**) Analogous (i) binds to four residues (ASPA:86, GLUA:51, LYSA:33, and ASPA:146 in the CDK1/CCNB1 complex. (**B**) Analogous (ii) binds to four residues (LYSA:88, GLNA:132, ASPA:146, and LYSA:33) in CDK1/CCNB1 complex. (**C**) Analogous (iii) binds to three residues (ASPA:146, LYSA:88, and GLNA:132) in the CDK1/CCNB1 complex. (**D**) Analogous (iv) binds to three residues (ASPA:86, GLUA:51, LYSA:33, and ASPA:146) in CDK1/CCNB1 complex. (**E**) Analogous (v) binds to two residues (ASPA:146 and GLUA:12) in CDK1/CCNB1 complex. (**F**) Analogous (vi) binds to three residues (LYSA:89, LYSA:88, and GLNA:132) in CDK1/CCNB1 complex. (**G**) Analogous (vii) binds to three residues (LYSA:89, GLNA:132, and LYSA:86) in CDK1/CCNB1 complex. (**H**) (viii) LT binds to three residues (LEUA:83, GLUA:12, and ASPA:146) in the CDK1/CCNB1 complex.

As illustrated in Figure 8A–H, LT analogues (i–viii) showed distinct binding interactions with the CDK1/CCNB1 complex under the applied docking protocol. Key residues involved included ASPA:86, GLUA:51, LYSA:33, ASPA:146, GLNA:132, LYSA:88, LYSA:89, GLUA:12, and LEUA:83. These interactions were mediated through hydrogen bonding, electrostatic forces, and hydrophobic contacts, with several residues consistently observed across analogues. Among the eight analogues, (v) showed the fewest hydrogen bonds. Taken together, the docking results are consistent with the possibility that LT analogues may interact with the CDK1/CCNB1 complex, and further experimental studies in ER^+^ BC models would be needed to explore their potential.

### 2.9. Network Pharmacology Identifies Luteolin Analogue Targeting of CDK1/CCNB1 as a Strategy to Suppress Breast Cancer Stem Cells

**Figure 9:**
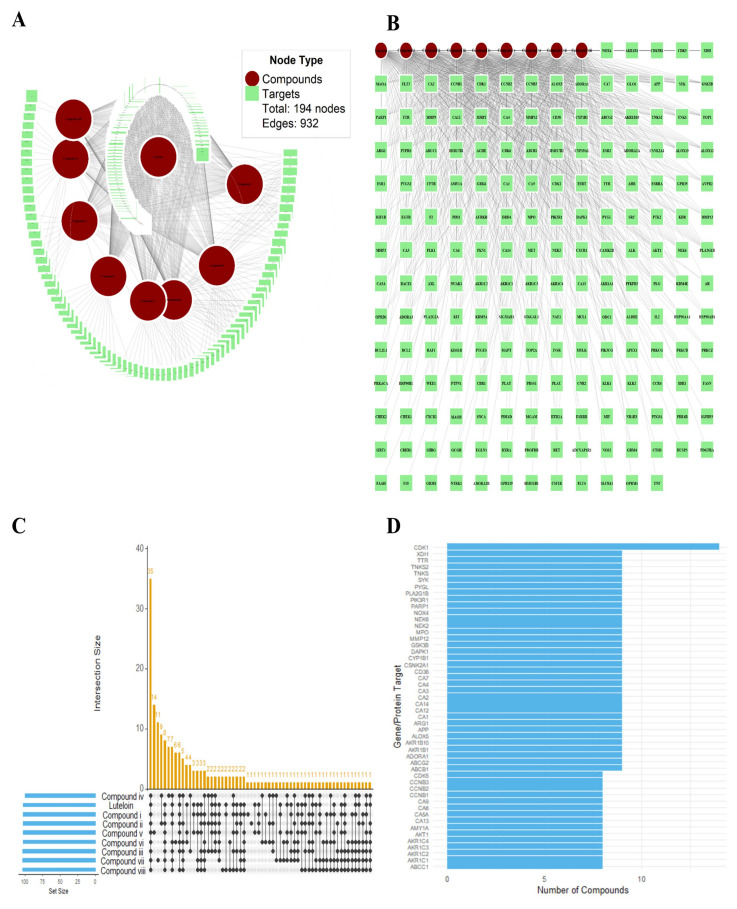

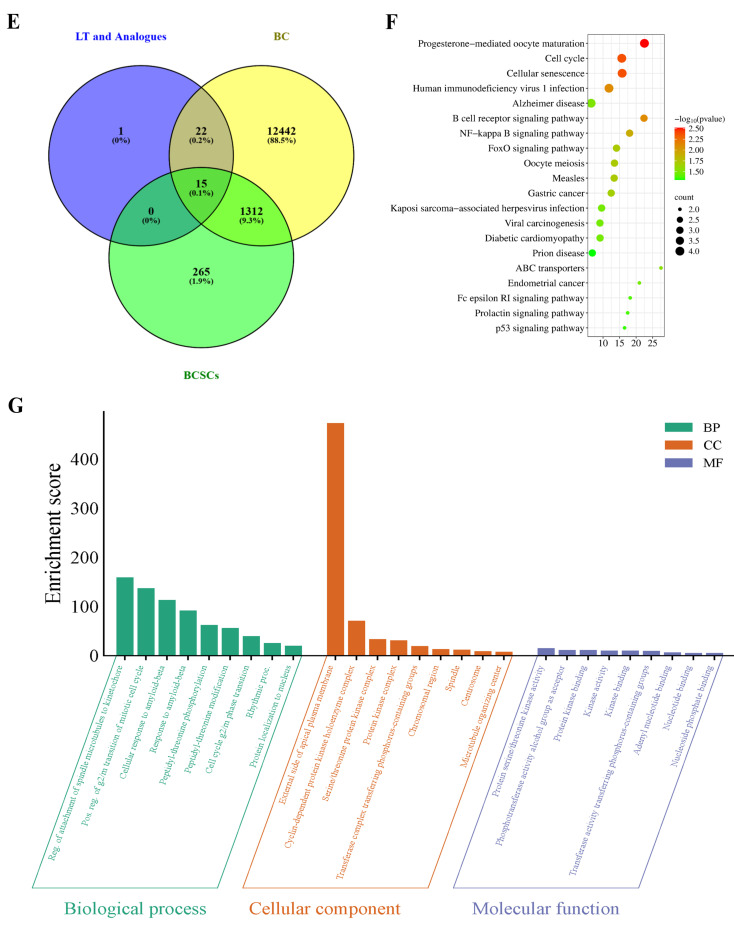

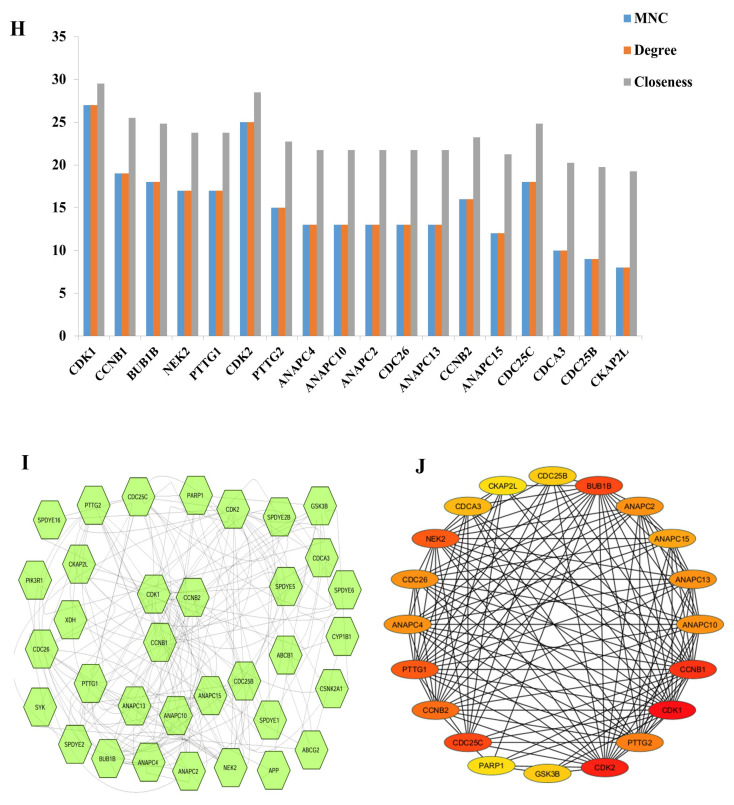
Network pharmacology and mechanistic profiling of LT and its analogues. (**A**) Radial tree diagram of the integrated compound target network. (**B**) Grid layout diagram of the integrated compound target network. (**C**) UpSetR plot illustrating the intersections of predicted gene/protein targets among LT and its eight structural analogues. Horizontal bars indicate the total number of targets per compound; vertical bars show the number of shared targets across specific compound subsets. This visualisation delineates unique and shared target profiles, forming the basis for comparative pharmacological analysis. (**D**) Horizontal bar chart ranking the 50 most frequently predicted gene/protein targets across all nine compounds. Bar length corresponds to the number of compounds predicted to target each gene, highlighting the most consistently implicated targets for downstream analysis. (**E**) A Venn diagram intersecting three target sets: (1) 38 predicted targets of eight LT analogue targets, (2) 13,791 breast cancer associated targets and (3) 1592 breast cancer stem cell essential targets. (**F**) Bubble chart of KEGG pathways significantly enriched among the predicted targets. Bubble size represents the number of targets in the pathway; colour intensity indicates the statistical significance of the enrichment. (**G**) GO analysis reveals shared gene functions. This graph displays the most significant MF, CC, and BP for the overlapping target genes. (**H**) Top-ranked hub proteins in the overlapping target genes (cytoHubba analysis). Degree represents the number of direct connections; Betweenness indicates control over information flow; MCC (Maximal Clique Centrality) measures robustness within cliques; Closeness reflects the efficiency of a node in reaching all other nodes in the network (inverse of average shortest path length). (**I**) Core PPI network of common targets. (**J**) Circular layout of the top 20 genes ranked by degree centrality. Nodes represent genes, with size and colour denoting topological importance (degree centrality).

To extend molecular insights to a systems level, a network pharmacology approach was employed. Potential protein targets for LT and its eight structural LT analogues were identified in silico using SwissTargetPrediction. The construction of an integrated compound–target network reveals the multi-target therapeutic potential of LT and its analogues (Figure 9A,B). The overlap of predicted targets was visualised using an UpSetR plot (Figure 9C). This analysis revealed both shared and unique protein targets among the compounds, offering insight into common pathways, particularly those relevant to the CDK1/CCNB1 complex. To identify the most prominent targets across the compound set, the 50 most frequently predicted gene/protein targets were selected. Their distribution is displayed in a horizontal bar chart (Figure 9D), where the y-axis lists gene names and the bar length indicates the number of compounds predicted to interact with each target. To elucidate the mechanisms underlying the anti-BC and anti-cancer stem cell effects of these compounds, we identified relevant disease targets. In our previous study, target proteins associated with breast cancer (BC) and BC stem cell populations were compiled from the GeneCards (17,002 targets) and OMIM (253 targets) databases, yielding a total of 17,255 targets. After removing duplicates, 13,791 unique targets with UniProt IDs were retained for BC, and a corresponding set of 1592 targets was defined for the BC stem cell population [10]. Thirty eight predicted targets of LT and its analogue, the intersection between these three target sets was identified using VENNY 2.1.0, revealing 15 common targets (Figure 9E). This intersection provides computational evidence that LT analogues may directly target genes essential for BC stem cell maintenance. KEGG pathway enrichment of these targets revealed significant association with ‘Progesterone-mediated oocyte maturation’ (*p* = 0.00304), ‘Cell cycle’ (*p* = 0.0042), and ‘Cellular senescence’ (*p* = 0.0042) (Figure 9F). ShinyGO 0.77 was used to annotate the 15 hub genes within the Gene Ontology frameworks of Biological Process (BP), Cellular Component (CC), and Molecular Function (MF). In the resulting plot, dot size represents the count of involved targets, visually prioritizing their significant involvement in the regulation of spindle microtubule-kinetochore attachment and the G2/M cell cycle transition (BP), their presence on the external apical plasma membrane and within cyclin-dependent kinase complexes (CC), and their protein serine/threonine kinase and phosphotransferase activities (MF) (Figure 9G). CDK1 and CCNB1 emerge as the most dominant nodes, exhibiting the highest degree, betweenness, MCC, and closeness centrality values, as summarized in Figure 9H. A protein–protein interaction network constructed using STRING indicated that CDK1 and CCNB1 forms a central hub connecting to neighbor proteins involved in cell cycle (Figure 9I). In Figure 9J, the circular layout illustrates the top 20 genes ranked by degree centrality in the interaction network with the highest degree, which is centered on CDK1 and CCNB1. Our designed LT analogue shared more than 85% of predicted targets with LT but showed higher predicted affinity for CDK family targets. Collectively, our integrated network pharmacology approach identifies the CDK1/CCNB1 axis as a central, targetable node, positioning LT analogue inhibition as a rational therapeutic strategy against BC stem cells.

### 2.10. Computational ADMET Profiling Reveals Optimal Drug-like Properties in Luteolin Derived Analogues

**Figure 10:**
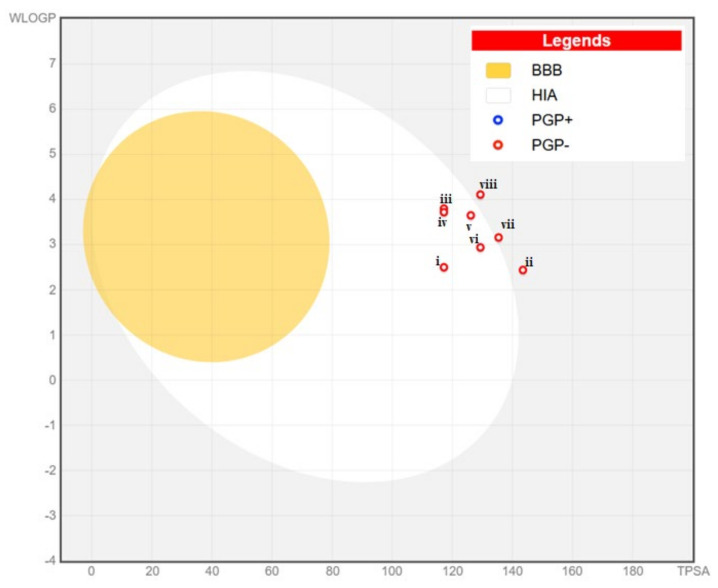
TPSA and WLOGP of top LT analogues plotted on the BOILED-Egg.

The ADMET analysis was used to determine a drug’s pharmacological and pharmacodynamic properties in the biological system [16]. Since LT analogues have physicochemical properties that enable oral bioavailability, they are promising candidates for drug development. Interestingly, these compounds are found in the white area of the egg plot, indicating better bioavailability profiles during a drug development phase, as shown in Figure 10, except for compounds ii, vii, and viii. This entire LT analogue does not cross the blood–brain barrier (Table 1), a protective barrier that separates the brain from circulating blood and prevents harmful substances from entering the brain. Additionally, these compounds have lower toxicity risks, making them safer for drug development (Table 1). LT analogues exhibit optimal Caco-2 permeability (Table 1), which is crucial for drug absorption in the human intestine. Caco-2 cells are widely used *in vitro* models for studying intestinal drug absorption and permeability [17]. These compounds have optimal permeability, allowing the human gut to absorb them effectively. This is a desirable characteristic for the development of new drugs. In addition, they exhibit a strong affinity for plasma proteins, which inhibits their passage through the blood–brain barrier, as indicated by positive HIA tests (Table 1). The half-life of the studied ligands indicates potential effectiveness (Table 1). Thus, Computational ADMET profiling confirms the optimized drug-like properties of these LT analogues, highlighting their promise as candidates for further development pending the necessary experimental validation of efficacy and safety.

Beyond absorption and distribution, the metabolic and excretion profiles of these LT analogues further support their drug development potential. As shown in Table 1, analogues i is the only LT analogue with no predicted interaction across all five major CYP isoforms, indicating a uniquely favorable metabolic profile, while others show varying isoform interactions. Regarding excretion, the predicted half-life (T1/2) values range from 0.659 to 1.363 h, with compound v exhibiting the longest half-life (1.363 h), while clearance values range from 1.122 to 8.552 mL/min/kg, with compounds vi and vii showing the lowest clearance (1.122 and 1.308 mL/min/kg, respectively), collectively suggesting prolonged systemic exposure and favorable elimination profiles across all LT analogues. All eight LT analogues satisfy Lipinski’s Rule of Five, confirming their drug-like physicochemical properties and supporting their suitability for oral drug development.

### 2.11. RMSF Analysis Reveals Luteolin Analogue-Specific Possible Allosteric Modulation of CDK1/CCNB1 Dynamics

**Figure 11:**
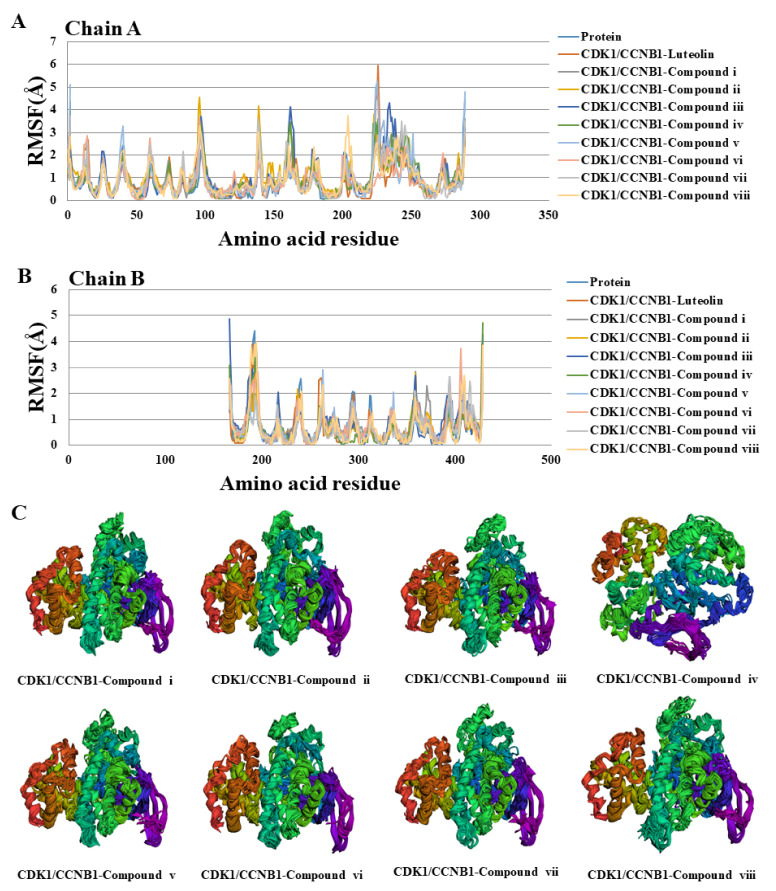
Molecular dynamics analysis of LT-induced flexibility changes (**A**) Comparative RMSF analysis of LT analogue in the CDK1/CCNB1 complex of Chain A. (**B**) Comparative RMSF analysis of LT analogue in the CDK1/CCNB1 complex of Chain B. (**C**) Ensemble of predicted structural models for the LT analogue in the CDK1/CCNB1 complex.

The RMSF analysis across all nine CDK1/CCNB1 complexes revealed a spectrum of ligand flexibility, with compound iii exhibiting the most pronounced dynamic profile under the simulation conditions, characterized by the highest mean (0.9538 Å) and maximum (4.8740 Å) fluctuations, along with the largest population of highly flexible atoms (19 > 3.0 Å). In contrast, compounds vi and vii showed the greatest overall stability within the simulated timescale, featuring the lowest mean fluctuations (0.8087 Å and 0.7898 Å, respectively) and the fewest highly mobile atoms (3 and 7 > 3.0 Å). The remaining compounds, including the LT complex, displayed intermediate flexibility, with mean values clustering around 0.82–0.92 Å and between 7–12 atoms exceeding 3.0 Å, as shown in Figure 11A,B. These observations are consistent with the presence of localized dynamic regions, while the structural integrity of each ligand within the binding site appeared to be generally maintained during the simulation. The panel presents the ten best computational models (Model 1 through Model 10) generated for the complex, displayed as a structural ensemble. Each model illustrates a possible three-dimensional conformation, with the protein shown in cartoon representation for all LT analogues; the CDK1/CCNB1 complexes are shown in Figure 11C. Analysis with CABS-flex 2.0 suggested that each LT analogue may modulate the dynamics of the CDK1/CCNB1 complex through distinct potential possible allosteric effects, although further investigations would be required to confirm these observations.

In summary, we leveraged network pharmacology to pinpoint the CDK1/CCNB1 complex as a druggable hub, then applied rational design to create bioavailable LT analogues that as revealed by molecular dynamics and ADMET profiling achieve potent and possible allosteric inhibition, presenting a comprehensive strategy to disrupt BC.

## 3. Discussion

Cancer remains one of the leading causes of mortality worldwide, and despite advances in targeted therapies, resistance and relapse remain major clinical challenges [18]. Among these challenges, acquired resistance to CDK4/6 inhibitors such as palbociclib and ribociclib has emerged as a growing clinical concern, often driven by cyclin E1 amplification or CDK2 reactivation, which bypasses G1 arrest [19]. The CDK1/CCNB1 axis is a central regulator of cell cycle progression, and its dysregulation is strongly associated with uncontrolled proliferation and poor prognosis in BC [3,20,21]. However, selective CDK1/CCNB1 inhibitors are limited, and the therapeutic potential of natural compounds to modulate this pathway remains underexplored. Notably, most existing CDK inhibitors target the ATP-binding pocket, leading to off-target effects and toxicity; our study diverges from this paradigm by identifying a natural flavonoid that may modulate complex stability rather than merely competing for the ATP site [22].

LT, a dietary flavonoid, has been reported to exert diverse anticancer effects. Yet, its direct molecular interactions with CDK1/CCNB1 and its roles in transcriptional and redox regulation remain insufficiently characterized [3,23]. Natural products continue to serve as a rich reservoir of anticancer agents, owing to their structural diversity and generally favourable toxicity profiles [24]. In this study, we identify LT, a dietary flavonoid, as a direct, high-affinity binder of the CDK1/CCNB1 complex, a critical regulator of the G2/M cell cycle transition [25]. Dysregulation of this complex is a hallmark of unchecked proliferation in various malignancies, including BC [26]. Addressing this gap was essential to determine whether LT could serve as a dual-function therapeutic agent, simultaneously suppressing cell cycle progression and modulating ROS levels. By integrating RNA-seq analysis, molecular modelling, and engineered cell line validation, this study provides mechanistic evidence that LT and its analogues directly target CDK1/CCNB1. These insights not only advance our understanding of LT’s anticancer activity but also lay the groundwork for natural product-inspired drug discovery, offering a novel strategy to selectively inhibit tumor growth while sparing normal cells [27]. A key mechanistic insight from our study is the discovery of a non-canonical redox–transcriptional axis, namely that LT suppresses ROS in non-malignant HEK293T cells but induces ROS in cancer cells [28], a duality that may underlie its selective toxicity [29]. This context-dependent behavior is rare for synthetic CDK inhibitors. It suggests LT could work as a ‘smart’ therapeutic by exploiting the elevated ROS levels common in cancer cells. Interestingly, LT reduced ROS at 15 μM but not at 30 μM in engineered HEK293T cells, revealing a concentration dependent switch. This narrow window deserves further investigation as a potential therapeutic strategy.

Our transcriptomic analyses across multiple datasets (GSE130262 [30], GSE71862 [31], and GSE130852 [32]) revealed that *CCNB1*, *CCNB2*, and *CDK1* are consistently downregulated in BC cells compared to non-malignant controls, underscoring tumor-specific regulation of these core cell cycle genes. This unexpected downregulation may reflect a feedback adaptation in established cancer cells or distinct regulatory mechanisms in malignant versus normal cycling cells, a point that merits further investigation in primary patient samples.

In contrast, our engineered HEK293T-GST-CDK1/CCNB1 cell line, designed to overcome negligible endogenous *CDK1* and *CCNB1* expression, showed that LT reduced ROS levels at 15 μM compared with 30 μM, diminished proliferation, and impaired inhibitory capacity compared with parental HEK293T cells. Notably, these phenotypic changes occurred with transcriptional impact on *CDK1* (minimal) and *CCNB1*, suggesting the existence of a feedback mechanism that modulates cell cycle progression under conditions of CDK1/CCNB1 overexpression. This feedback loop potentially involving p53, p21, or post-translational modifications such as CDK1 phosphorylation represents an important area for future mechanistic dissection [33]. To directly address any conceptual concern, we clarify that “ROS reduction” applies to HEK293T-derived cells, while “oxidative stress induction” is not claimed for these cells but rather occurs specifically in cancer cell lines. These are context-dependent, non-contradictory outcomes. Taken together, these results emphasise the dual nature of LT’s activity, functioning as a ROS suppressor in non-cancerous contexts and as a prooxidant in malignant environments. This selective, context-dependent action, driven by cellular redox and signalling interplay, positions LT as a promising therapeutic that could target cancer cells while sparing normal cell. Molecular modeling and molecular dynamic analyses provide compelling evidence that LT establishes stable, favourable interactions with the CDK1/CCNB1 complex. The LT and its analogue’s dynamic engagement within the binding pocket suggests a flexible binding mode that may destabilise the complex and impair its regulatory function. Unlike ATP inhibitors that block the active site, our simulations suggest LT binds elsewhere near the cyclin interface. That probably disrupts CDK1/CCNB1 dimerization or locks it inactive. This allosteric-like action might explain why LT could be more selective than ATP mimics, which often cross-react with other CDKs [34]. Such disruption likely contributes to the observed transcriptional repression of *CDK1* and *CCNB1*, reinforcing LT’s role as a modulator of cell cycle machinery. Collectively, these findings position LT as a promising candidate for selective inhibition of the CDK1/CCNB1 complex. Notably, the analogue 5-O-acetyl LT indicated particularly high binding affinity toward CDK1/CCNB1, further supporting the therapeutic potential of this chemical class. The LT analogues designed through our computational pipeline indicate strong, stable binding to the CDK1/CCNB1 complex. This target is corroborated by network pharmacology analysis, which identifies cell cycle regulation in BC stem cells as the primary affected pathway. The convergence on BC stem cells is particularly significant, as these cells are thought to drive relapse and chemoresistance; LT’s ability to target stemness-associated pathways suggests potential utility in adjuvant or maintenance settings to prevent recurrence [35]. The convergence of LT and its analogues on this key node, supported by their favourable ADMET profiles combined with its flavonoid scaffold, provides an attractive foundation for further medicinal chemistry optimization [36] and enhanced docking interactions, warranting further evaluation in models such as MCF7. Future medicinal chemistry efforts, including rational modifications at the 5- and 7-hydroxyl positions, could be guided by our docking simulations to improve binding affinity and specificity for the complex interface [37].

We acknowledge that HEK293T cells are not representative of BC biology. Therefore, while our findings provide preliminary mechanistic insights, comprehensive validation in MCF-7 and TNBC BC cell lines is necessary before extending conclusions to BC. Our integrated experimental and computational pipeline predicts that rational, structure-guided modification of LT can yield analogues that selectively target the CDK1/CCNB1 axis, exploiting the redox-transcriptional vulnerability identified here. Together, these insights solidify LT’s role as a context-dependent modulator of cell-cycle regulation and position it as a candidate for precision therapeutic development in BC. These findings have immediate implications. First, they advocate shifting screening strategies for CDK1 inhibitors beyond ATP-pocket competitors. Targeting complex stability or sensitizing the complex to endogenous stress pathways offers a promising route to greater selectivity [38]. Second, LT and its optimized analogues could serve as lead compounds for pro-oxidant cancer therapy, particularly in tumors with known redox vulnerabilities. Third, the CDK1/CCNB1 “barrier” signature may have diagnostic or prognostic value in identifying tumors that rely on alternative cyclin complexes.

### Limitations and Future Directions

Our computational docking and MD simulations alone are insufficient to definitively prove allosteric inhibition. Direct experimental validation, such as site-directed mutagenesis of predicted binding residues, binding pocket comparisons with known allosteric inhibitors, or biophysical methods (SPR, ITC), is required before concluding an allosteric mechanism. Moreover, we could not perform Western blot or fluorescence microscopy to verify stable expression of the HEK293T-GST-CDK1/CCNB1 cell line, as these specific validation methods were not available in our setting. GAPDH expression can vary under certain conditions (e.g., hypoxia, metabolic stress, drug treatment), which may affect normalization accuracy. Future studies should validate key findings using additional reference genes (e.g., β-actin, 18S rRNA, or multi-gene normalization). Stemness-related findings are derived from network pharmacology predictions, not direct experimentation. Validation using stemness markers (CD44, CD24, ALDH1A1) and functional assays (mammosphere formation, limiting dilution) is required. These experiments were beyond the current scope and will be addressed in future work. This study primarily establishes mechanisms in a single, engineered HEK293T-GST-CDK1/CCNB1 cell line. Thus, our findings are best viewed as a hypothesisgenerating foundation, not definitive mechanistic proof. Nevertheless, we establish a robust biochemical and cellular foundation for advancing LT-based CDK1/CCNB1 inhibitors, reinforcing the promise of natural product-inspired strategies in cancer therapeutics. Future investigations should prioritize the following: (i) SPR or CETSA to validate LT–CDK1/CCNB1 binding; (ii) *in vivo* testing of 5-O-acetyl LT in xenograft models; (iii) co-crystallography to resolve the binding mode; (iv) genetic perturbation studies to establish causality; (v) validation in patient-derived BC organoids or xenograft models for translational relevance; and (vi) biochemical validation of allosteric inhibition *via* CDK1 enzyme activity assays and pull-down/Co-IP experiments in relevant cancer cell lines beyond the HEK293T proof-of-concept model. We believe these next steps will determine whether LT analogues merit further development as selective CDK1 inhibitors for BC [39]. Together, these insights highlight LT and its derivatives as compelling leads for the development of novel CDK1/CCNB1 inhibitors, with clear potential for targeted cancer therapy.

## 4. Materials and Methods

### 4.1. Expression Profiling of CDK1, CCNB1 and CCNB2 Across HEK293T, MCF10A, and MCF7

Expression profiles of *CCNB1*, *CCNB2*, and *CDK1* genes in MCF-7 BC cell lines and HEK293T cells were obtained from the Gene Expression Omnibus (GEO) and validated through qPCR. MCF-7 cells were cultured to 70–80% confluency before RNA extraction. Total RNA was isolated using TriQuick Reagent (Solarbio, cat# R1100, Beijing, China), followed by chloroform extraction and isopropanol precipitation. The RNA pellet was washed, air-dried, and resuspended in DEPC-treated water. cDNA synthesis was performed using the StarScript III-in-one RT mix with gDNA Remover (GeneStar, cat# A230-10, Beijing, China) cDNA Synthesis Kit. RNA and cDNA concentrations were measured with a Nanodrop spectrophotometer (N60/N50 Nanophotometer, IMPLEN, Munich, Germany). Quantitative RT-PCR was performed using 2 × RealStar Fast SYBR qPCR Mix (Gene Star, cat# A311-10, Beijing, China) according to the manufacturer’s protocol. Each sample was examined in triplicate, and Ct values were normalised to GAPDH. Delta Ct values plotted to compare relative expression levels.

Additionally, comprehensive gene expression data for *CCNB2*, *CCNB1*, and *CDK1* across various BC cell lines were retrieved from the Expression Atlas. To further investigate expression dynamics, we retrieved publicly available transcriptomic datasets from the Gene Expression Omnibus (GEO): GSE130262 (HEK293T), GSE71862 (MCF10A [control] and MCF7 [cancer]), and GSE130852 (MCF7). We downloaded the RNA-seq datasets from the NCBI Sequence Read Archive (SRA) using the SRA Toolkit. After retrieval, we carried out an initial preprocessing step to ensure data quality. Specifically, we evaluated raw sequencing reads with FASTQC, which enabled us to inspect key quality metrics, including per-base sequence quality, GC content, sequence duplication levels, and the presence of overrepresented sequences. Based on these quality reports, we used trimmomatic to trim reads and remove adapter contamination and low-quality bases, thereby improving the reliability of downstream analyses. Following preprocessing, we aligned the cleaned reads from each sample to the human reference genome using the STAR aligner. STAR was selected for its speed and accuracy in mapping spliced reads, which are critical for transcriptomic data. The resulting alignment files (BAM format) were then processed to quantify gene expression. For this step, we used the feature counts tool to assign aligned reads to annotated genes and generate a count matrix containing raw read counts for all genes across all samples. Each GEO dataset (GSE130262, GSE71862, and GSE130852) was processed and analyzed independently. Normalized expression values of CDK1, CCNB1, and CCNB2 were subsequently extracted for comparative visualization across the different cell lines.

We then conducted differential gene expression analysis using DESeq2 in R. The count matrix was imported into DESeq2, normalised to account for differences in sequencing depth and RNA composition across samples, and statistically modelled to identify genes with significant expression changes between experimental conditions. After obtaining the differential expression results, we focused on three genes of particular interest. We extracted their normalised expression values from the full dataset and filtered them to retain only these genes across all samples for targeted comparison. Finally, all visualisations, including quality summaries, expression plots, and differential expression figures, were generated using custom R scripts.

### 4.2. Cell Culture

MCF7 (RRID: CVCL_0031) and HEK 293T (RRID: CVCL_0063) cell lines were obtained from our institute’s cell bank (Guangzhou Institutes of Biomedicine and Health, Chinese Academy of Sciences, Guangzhou, China). All cell lines used in this study were routinely authenticated and checked for mycoplasma contamination. HEK293T cells were grown in DMEM (Gibco, cat# C11995500BT, Suzhou, China). The media were supplemented with 10% FBS (Viva Cell Biosciences, cat# C04001-500, Shanghai, China) and 1% penicillin–streptomycin (Gibco, lot#1924793, New York, NY, USA). All reagents were of molecular biology grade. The cells were incubated at 37 °C in a humidified atmosphere with 5% CO_2_.

### 4.3. Cell Viability Studies

HEK 293T cells were seeded at 5000–6000 cells/well in 96-well plates and incubated for 24 h at 37 °C with 5% CO_2_. LT dissolved in DMSO (final concentrations: 40 μM to 10 μM, with 0.5% DMSO) was added to the cell culture, followed by a 48 h incubation. Then, 10 μL of CCK-8 solution (Biosharp Life Science, cat# BS350A, Hefei, Anhui Province, China) was added to each well, and the plates were incubated for 2–4 h at 37 °C. Absorbance was measured at 450 nm using the Epoch 12 microplate reader. *Cell Inhibition was calculated using Equation (2)*.

*Cell Viability* (%) = [(*OD_exp* − *OD_blank*)/(*OD_control* − *OD_blank*)] × 100 (1)

*Cell Inhibition* (%) = 100 − *Cell Viability* (%) (2)

OD_exp_ is the absorbance of the sample, OD_blank_ is the absorbance of the blank, and OD_control_ is the absorbance of the control sample.

### 4.4. Chemical Transformation of Competent Cells

Two microliters of pre-chilled pCMV-GST-CDK1 plasmid (human-Neo plasmid) (Miaoling plasmid P50658, Wuhan, China) were added to 100 μL of pre-chilled chemically competent cells (DH5α BC102) in a 1.5 mL microcentrifuge tube. The mixture was gently homogenised and incubated on ice for 30 min, followed by heat shock at 42 °C for 60 s and immediate cooling on ice for 2 min. Transformed cells were resuspended in 900 μL of pre-warmed, antibiotic-free LB medium and recovered at 37 °C, 180 rpm for 45 min. Cells were pelleted at 6000 rpm for 3 min, resuspended in 100 μL of supernatant, and plated onto LB agar containing ampicillin. Colonies were cultured overnight at 37 °C and screened by colony and plasmid PCR.

### 4.5. Transfection of PCMV-GST-CDK1 Plasmid into HEK293T Cells

HEK293T cells were cultured in DMEM supplemented with 10% FBS and 1% penicillin–streptomycin at 37 °C in a humidified incubator with 5% CO_2_. For transfection, cells were seeded at 2 × 10^5^ cells/well in 6-well plates and incubated overnight to reach 70–80% confluence. The medium was replaced with antibiotic-free DMEM containing 10% FBS before transfection. pCMV-GST-CDK1 (human)-Neo plasmid (2–3 μg) was transfected using StarFect Lip2000 Transfection Reagent (GeneStar, cat# C105-01, Beijing, China), following the manufacturer’s protocol. DNA and lipofectamine were separately diluted in Opti-MEM (Gibco, cat# 31985-070, New York, NY, USA), incubated for 5 min, combined, and incubated for 20 min to form complexes. The 100 μL transfection mixture was added dropwise to each well. After 8–10 h, the medium was replaced with fresh DMEM containing 10% FBS and 1% penicillin–streptomycin.

### 4.6. Generation of HEK293T-GST-CDK1/CCNB1 Cell Line

Cells were initially transfected with pCMV-GST-CDK1 (human)-Neo using Lipofectamine 2000 and cultured for 24–48 h. Selection was initiated with DMEM supplemented with 10% FBS and 600 μg/mL G418 (MP Biomedicals, cat# 158782, Santa Ana, CA, USA), and maintained for 8–10 days with medium changes every 2–3 days. To enhance expression, cells were retransfected with the same plasmid using Lipofectamine 8000 (Beyotime, cat# C0533, Shanghai, China) and subjected to an additional 4–6 days of selection at 1000 μg/mL G418. Resistant colonies were expanded, and GST-CDK1 expression was confirmed by qPCR analysis as described in Section 4.1.

### 4.7. Quantitative RT-PCR Analyses

HEK293T and expressed HEK293T cells were incubated in the proper medium until they reached 70–80% confluency. Cells were then divided into control (untreated) and LT-treated groups, with MCF-7 cells treated with 15 µM and 30 µM LT for 46–48 h. Vehicle controls were included. Total RNA was isolated using TriQuick Reagent (Solarbio, cat# R1100, Beijing, China), followed by chloroform extraction and isopropanol precipitation. The RNA pellet was washed, air-dried, and resuspended in DEPC-treated water. cDNA synthesis was performed using the StarScript III-in-one RT mix with gDNA Remover (GeneStar, cat# A230-10, Beijing, China) cDNA Synthesis Kit. RNA and cDNA concentrations were measured with a Nanodrop spectrophotometer (N60/N50 Nanophotometer, IMPLEN, Munich, Germany). Quantitative RT-PCR was performed using 2 × RealStar Fast SYBR qPCR Mix (Gene Star, cat# A311-10, Beijing, China) according to the manufacturer’s protocol. Each sample was examined in triplicate, and Ct values were normalised to *GAPDH*. Each sample was examined in triplicate, and relative mRNA expression levels were determined using the 2^^ΔΔCq^ method, normalized to the housekeeping gene *GAPDH*. Sangon Biotech (Shanghai, China) synthesised the primers. The primers used for qPCR are shown in Table 2.

### 4.8. ROS Determination

Expressed HEK293T cells were propagated in the recommended growth medium and allowed to reach approximately 70–80% confluency. Cells were divided into two groups: a control group and an LT-treated group. ROS determination was performed by using 2′,7′-Dichlorodihydrofluorescein diacetate (DCFH-DA) staining to see the effect of LT on ROS production. The MCF-7 cells were briefly treated with 15 µM and 30 µM of LT and vehicle control (DMSO) for 46–48 h. After the stipulated treatment time, cells were further incubated with 10 Mm DCFH-DA (Beyotime, cat# S0033, Shanghai, China) for 20–25 min. The cells were trypsinised, washed twice with PBS, and analysed for ROS production using CytoFLEX flow cytometry (Beckman Coulter, Brea, CA, USA). Data were exported to FCS files and analysed with FlowJo v10.8.1.

### 4.9. Pharmacophore Modeling of Luteolin Analogues

A ligand-based pharmacophore model was generated in Maestro (Schrödinger). The 3D structures of LT and its analogues were prepared and minimised using the standard preparation protocol in Maestro. A single-ligand workspace was created with LT, and its analogues as the active ligand, and pharmacophore features were automatically detected. From the generated features, hydrogen bond acceptor sites (A) and hydrophobic features (H) were selected based on their relevance to putative binding interactions. These features were used to construct the final pharmacophore hypothesis, representing the key interaction pattern required for activity. The resulting pharmacophore model was then used to analyse LT analogues for their ability to map onto the selected features and preserve the essential pharmacophoric arrangement.

### 4.10. Prediction of Drug-likeness, Biological Activity, and ADMET Analysis of Luteolin Analogue

The SMILES notations of all LT analogues were submitted to the MolSoft platform (https://www.molsoft.com/mprop, accessed on 1 December 2025) to predict drug-likeness scores and key physicochemical properties. Subsequently, the SMILES notations of all LT analogues were submitted to PASS Online (https://way2drug.com/PassOnline/predict.php, accessed on 10 December 2025) to predict probable biological activities, with emphasis on anti-BC activity [40]. Finally, potential molecular targets were identified, using SwissTargetPrediction (https://www.swisstargetprediction.ch/, accessed on 19 December 2025) using the same SMILES inputs. The ADMET pharmacokinetic properties primarily address the toxicity, excretion, metabolism, distribution, and absorption of ligands in the human body. The ADMET profile was evaluated using the ADMETsar server (http://lmmd.ecust.edu.cn/admetsar2/, accessed on 26 December 2025) [41], the Swiss ADME server (http://www.swissadme.ch/index.php, accessed on 26 December 2025) [42], and the ADMETLab server (https://admetmesh.scbdd.com/service/evaluation/cal, accessed on 27 December 2025) [43].

### 4.11. Molecular Docking and Dynamics Study of Luteolin and Its Analogue with CDK1/CCNB1

Molecular docking was performed as previously described [29]. The CDK1/CCNB1 complex (PDB: 4Y72) was prepared by removing water molecules, adding hydrogen atoms and assigning Kollman charges. Docking grids were centred on the ATP-binding site (dimensions: 20 × 20 × 20 Å). Docking simulations were conducted with an exhaustiveness parameter of 8. The best pose was selected based on binding energy. All computational studies were performed using the Schrödinger software suite (v2021-3). The Desmond package was used for Root Mean Square Deviation (RMSD) and Root Mean Square Fluctuation (RMSF) analyses for LT. Additionally, the conformational dynamics of the CDK1/CCNB1 complex for LT and its analogue were simulated using the CABS-flex 2.0 coarse grained modeling procedure, with RMSF analysis.

### 4.12. Molecular Mechanics/Generalised Born Surface Area (MM-GBSA) Calculations

Binding free energies were estimated using the prime MM-GBSA module in Schrödinger Suite. Protein ligand complexes were prepared with the Protein Preparation Wizard, assigning protonation states with Epik at pH 7.0 ± 2.0. Ligands were minimised using the OPLS4 force field. MM-GBSA calculations were performed with the VSGB 2.1 implicit solvent model, a refit of VSGB 2.0. The workflow involved minimising the receptor, ligand, and complex, followed by extracting receptor and ligand energies from the minimised complex.

*Binding free energies (ΔG_bind) were calculated as*

*ΔG*_*bind*_ = *E*_*Complex*_ − (*E*_*Receptor*_ + *E*_*Ligand*_) (3)

Strain-corrected binding energies (ΔG_bind(NS)) were obtained by subtracting receptor and ligand strain contributions. Energies were decomposed into Coulombic, van der Waals, covalent, lipophilic, solvation, hydrogen bonding, packing, and self-contact terms. The data were analysed using RGui (R version 4.5.2).

### 4.13. Network Pharmacology Analysis

The canonical SMILES structures of LT and eight structural analogues, along with their potential protein targets in *Homo sapiens*, were predicted using SwissTargetPrediction. The data were analysed using RGui (R version 4.5.2), and associated genes for BC and BC stem cells were compiled from GeneCards and OMIM, and standardised. Redundant entries were removed to merge all acquired targets. The intersection of compound predicted targets and disease-related genes was identified using VENNY 2.1.0. The resulting common targets were functionally characterised *via* Gene Ontology (GO) and KEGG pathway enrichment analyses using ShinyGO 0.77 (FDR < 0.05). A protein–protein interaction (PPI) network was constructed from the STRING database (confidence score > 0.4) and analysed with Cytoscape (v3.9.1) and the CytoHubba plugin to identify hub genes.

### 4.14. Statistical Analyses

All analyses were performed using GraphPad Prism software (version 9) and RGui (R version 4.5.2). Data were analyzed using one-way ANOVA (Brown–Forsythe and Welch tests) and are presented as mean ± SD. All experiments were performed with at least three independent biological replicates; within each biological replicate, three technical replicates were measured, and their mean was used as a single data point. Exact *p*-values are reported alongside significance indicators. A *p*-value < 0.05 was considered statistically significant: * *p* < 0.05, ** *p* < 0.01, *** *p* < 0.001, **** *p* < 0.0001. Error bars in all figures represent SD.

## 5. Conclusions

In summary, direct evidence from engineered HEK293T cells expressing the GST-tagged CDK1/CCNB1 complex suggests that LT binds to and interferes with this kinase complex. LT appears to inhibit CDK1/CCNB1 activity, which may lead to transcriptional modulation and changes in ROS levels. These molecular alterations may contribute to reduced cancer cell proliferation and have a possible effect on stemness properties based on network pharmacology. Collectively, these findings suggest that CDK1/CCNB1 could be a therapeutically relevant target in cancers driven by this dysregulated axis. Moving forward, rational structure-based development of LT analogues, coupled with advanced delivery strategies, may hold promise for achieving the selective disruption of cell-cycle progression in such malignancies, though further validation is needed.

## Figures and Tables

**Table 1:** Lists the pharmacokinetic and toxicity properties of the LT analogue.

Characteristics			CDK1/CCNB1 Inhibitors						
		(i)	(ii)	(iii)	(iv)	(v)	(vi)	(vii)	(viii)
**Lipid Solubility**									
WLOGP		2.50	2.44	3.80	3.72	3.65	2.94	3.16	4.11
Consensus Log *P*_o/w_		1.88	1.94	3.06	3.09	2.51	2.79	2.96	3.80
**Aqueous Solubility**									
ESOL Logs		−3.49	−3.43	−4.91	−4.88	−3.84	−4.28	−4.09	−5.20
ESCOL Class		Soluble	Soluble	Moderately soluble	Moderately soluble	Soluble	Moderately soluble	Moderately soluble	Moderately soluble
**Absorption and Distribution**									
BBB Permeant		No	No	No	No	No	No	No	No
Bioavailability Score	OB ≥ 0.3	0.55	0.55	0.55	0.55	0.55	0.55	0.55	0.55
Plasma Protein Binding (PPB)	Optimal: <90%. The drugs with high protein binding may have a lower therapeutic index	90.19%	88.40%	99.98%	98.85%	98.007%	80.76%	77.28%	92.35%
Human Intestinal Absorption (HIA)	HIA + (HIA < 30); HIA − (HIA ≥ 30)	0.033	0.2	0.022	0.022	0.017	0.45	0.615	0.47
Volume Distribution (VD)	Optimal: 0.04–20 L/kg	0.61	0.537	0.424	0.405	−0.561	0.519	0.632	0.353
MDCK permeability	Low: <2 × 10^6^, Medium: 2–20 × 10^6^; High: >20 × 10^6^ cm/s	1.5 × 10^−5^	1.8 × 10^−5^	1.6 × 10^−5^	1.4 × 10^−5^	−4.798	6.2 × 10^−5^	3.2 × 10^−5^	3 × 10^−5^
Caco-2 permeability	Optimal: Higher than −5.15 log unit	−4.882	−4.799	−4.97	−4.96	−5.306	−4.835	−4.874	−4.989
**Metabolism**									
CYP1A2		No	Yes	No	No	Yes	Yes	Yes	Yes
CYP2C19		No	No	No	No	No	No	No	Yes
CYP2C9		No	Yes	Yes	Yes	No	Yes	Yes	Yes
CYP2D6		No	No	Yes	Yes	Yes	No	No	No
CYP3A4		No	No	No	No	Yes	Yes	Yes	Yes
**Excretion**									
T_1/2 (h)_		0.908	0.948	0.912	0.913	1.363	0.686	0.659	0.716
Clearance (ml/min/kg)		4.159	8.552	6.401	8.054	7.893	1.122	1.308	4.268
**Toxicity**									
hERG blocker	1: active; 0: inactive	0.008	0.006	0.02	0.021	0.024	0.001	0.0	0.002
Rat Oral Acute Toxicity (ROAT)	0: Low toxicity; 1: High toxicity	0.08	0.049	0.058	0.62	0.658	0.641	0.631	0.351
Human Hepatic Toxicity	1: H-HT positive; 0: H-HT negative	0.033	0.081	0.037	0.04	0.006	0.003	0.001	0.003
Respiratory toxicity	1: Respiratory toxicants; 0: Respiratory non-toxicants	0.266	0.121	0.178	0.146	0.682	0.24	0.111	0.214
Carcinogenicity	1: Carcinogens; 0: Non-carcinogens	0.105	0.045	0.16	0.195	0.76	0.115	0.089	0.106
**Drug-like property**									
Lead likeliness	250 ≤ MW ≤ 350; XLOGP = 3.5; Num. of rotatable bonds ≤ 7	Yes	No, 1 violation: MW > 350	No; 2 violations: MW > 350, XLOGP3 > 3.5	No; 2 violations: MW > 350, XLOGP3 > 3.5	Yes	No, 1 violation: MW > 350	No; 2 violations: MW > 350, Rotors > 7	No; 3 violations: MW > 350, Rotors > 7, XLOGP3 > 3.5
Drug likeness (Lipinski rule)	MW ≤ 500; MLogP ≤ 5; N or O ≤ 10; NH or OH ≤ 5	Accepted	Accepted	Accepted	Accepted	Accepted	Accepted	Accepted	Accepted

**Table 2:** Primer sequences in RT-qPCR and plasmid verification.

Gene	Description	Sequence (5′-3′)
*CCNB1*	Forward Sequence	GACCTGTGTCAGGCTTTCTCTG
	Reverse Sequence	GGTATTTTGGTCTGACTGCTTGC
*CCNB2*	Forward Sequence	CAACCAGAGCAGCACAAGTAGC
	Reverse Sequence	GGAGCCAACTTTTCCATCTGTAC
*CDK1*	Forward Sequence	GGAAACCAGGAAGCCTAGCATC
	Reverse Sequence	GGATGATTCAGTGCCATTTTGCC
*GAPDH*	Forward Sequence	GTCTCCTCTGACTTCAACAGCG
	Reverse Sequence	ACCACCCTGTTGCTGTAGCCAA
pCMV-GST-CDK1 (human)-Neo plasmid	Forward Sequence	ATGGACCCAATGTGCCTGGATG
	Reverse Sequence	CCACATAGCGTAAAAGGAGCAAC

## Data Availability

The original contributions presented in this study are included in the article. Further inquiries can be directed to the corresponding author.

## Abbreviations

BC	Breast Cancer
BP	Biological Process
CC	Cellular Component
CCNB1	Cyclin B1
CCNB2	Cyclin B2
CDK1	Cyclin-Dependent Kinase 1
LT	Luteolin
MF	Molecular Function
RMSD	Root Mean Square Deviation
RMSF	Root Mean Square Fluctuation
